# A joint design for functional data with application to scheduling ultrasound scans^☆^

**DOI:** 10.1016/j.csda.2018.01.009

**Published:** 2018-06

**Authors:** So Young Park, Luo Xiao, Jayson D. Willbur, Ana-Maria Staicu, N. L’ntshotsholé Jumbe

**Affiliations:** a Eli Lilly and Company, Indianapolis, IN, USA; b North Carolina State University, Raleigh, NC, USA; c Metrum Research Group LLC, Tariffville, CT, USA; d Bill & Melinda Gates Foundation, Seattle, WA, USA

**Keywords:** Covariance function, Functional data analysis, Fetal growth, Longitudinal data, Prediction

## Abstract

A joint design for sampling functional data is proposed to achieve optimal prediction of both functional data and a scalar outcome. The motivating application is fetal growth, where the objective is to determine the optimal times to collect ultrasound measurements in order to recover fetal growth trajectories and to predict child birth outcomes. The joint design is formulated using an optimization criterion and implemented in a pilot study. Performance of the proposed design is evaluated via simulation study and application to fetal ultrasound data.

## 1 Introduction

Functional data analysis has been a popular statistical research area for the last two decades and has found application in many fields such as brain imaging Jiang et al. (2009), Greven et al. (2010), Reiss and Ogden (2010), Lindquist (2012), Lu and Marron (2014), Park and Staicu (2015), biosignals Crainiceanu et al. (2012), Randolph et al. (2012), Goldsmith and Kitago (2016), genetics Tang and Müller (2009), Reimherr and Nicolae (2014) and wearable computing Morris et al. (2006), Li et al. (2014), Xiao et al. (2015). For a comprehensive treatment of functional data analysis see Ramsay and Silverman (2002), Ramsay and Silverman (2005) and Horváth and Kokoszka (2012).

This paper considers sampling design for noisy growth data. The motivation arises from the study of fetal growth, where measurements of fetal size may be obtained during pregnancy using ultrasound. And the particular question to be addressed is: when a fixed number of ultrasound scans will be taken during pregnancy, what are the optimal time points for data collection? Optimality can be defined either in terms of recovering individual fetal growth trajectories or in terms of predicting a birth outcome, such as birth weight. However, in practice it may be important to predict both individual growth trajectories and birth outcomes, and in such cases a joint optimality criterion must be formulated. We also consider the closely related question of the number of ultrasound scans required to achieve a desired level of optimality.

We address this question within the functional data framework. Design for functional data has received some interest recently. For example, Ferraty et al. (2010) considered a nonparametric model with a scalar response and a functional predictor and Delaigle et al. (2012) studied a similar problem for classifying and clustering functional data. Both methods are restricted to densely sampled functional data and focus on dimensionality reduction for a dense functional predictor. And for spatially correlated functional data, Rasekhi et al. (2014) and Bohorquez et al. (2015) considered the problem of selecting spatial sampling points.

Design for functional data has also been extended to longitudinal data. Ji and Müller (2017) proposed prediction-based criteria for sampling functional data with the target of either recovering individual functions or predicting a scalar outcome. Wu et al. (2017) exploited the mixed effects model representation of functional data and proposed a design criterion based on Fisher’s information matrix of eigenvalues of the covariance function. There are several limitations with these approaches. Wu et al. (2017) focused on recovering individual functions, while Ji and Müller (2017) were limited to the study of design separately and did not consider a joint design, which is the focus of our data application. In addition, in these works the number of design points was pre-fixed and no data-driven method was developed. Finally, Ji and Müller (2017) did not compare functional data models versus parametric mixed effects models for prediction-based designs. Our work addresses these gaps.

Following early work on design, such as Ylvisaker (1987) and the references therein and recent work by Ji and Müller (2017), we consider prediction-based designs and propose a unified design criterion for both recovering individual functions as well as predicting scalar outcomes from a functional predictor. We also propose a practical data-driven method for selecting the number of design points, building on the result that the larger the number of design points, the better the prediction will be (see Theorem 1). Finally we conduct a comprehensive simulation study to evaluate the performance of functional data models as compared to parametric mixed effects models, and demonstrate numerically that functional data models might be preferred over parametric mixed effects models for prediction-based optimal designs for longitudinal data.

The rest of the paper is organized as follows. In Section 2 we introduce functional data models and propose a unified prediction-based design criterion for sampling functional data. In Section 3 we study the theoretic properties of the proposed design. In Section 4 we discuss implementation of the design and propose a data-driven method for selecting the number of design points. In Section 5 we illustrate the proposed method using a fetal ultrasound data. In Section 6, we investigate the performance of the design via simulation studies.

## 2 Optimal design for functional data

In this section, we first describe functional data models and then formulate two optimal design problems for sampling functional data: one design targets accurate prediction of individual functions while the other targets accurate prediction of a scalar outcome. Then, we propose a unified design criterion that targets both recovering individual functions and predicting a scalar outcome. In particular, the unified design contains the previous two designs as special cases.

### 2.1 Statistical models

Consider a random function X(t)(t∈T) defined over a continuous and compact time domain T. Suppose that X(⋅) is a Gaussian process with mean function μ(t)=E{X(t)} and covariance function r(s,t)=Cov{X(s),X(t)}. We assume that X(⋅) is square integrable in T and without loss of generality we let T=[0,1].

In practice, X(⋅) is observed at a finite number of time points and contaminated with noise. Hence, for a random function $X_{i}\left(\cdot \right)$ with a subject index i observed at p time points ${\left(t_{1},\ldots ,t_{p}\right)}^{\prime }\in T^{p}$, the observations are

(1) $$W_{ij}=X_{i}\left(t_{j}\right)+\epsilon_{ij},\;\;1\leq j\leq p,$$

where the $\epsilon_{ij}$ are i.i.d. $N\left(0,\sigma_{\epsilon }^{2}\right)$ and independent of $X_{i}\left(\cdot \right)$.

Let Y be a scalar outcome with a functional predictor X(⋅). And consider the functional linear model

(2) $$Y=\alpha +\int_{T}\overset{¯}{X}\left(t\right)\beta \left(t\right)dt+e,$$

where α is an intercept, $\overset{¯}{X}\left(t\right)=X\left(t\right)-\mu \left(t\right)$, β(t) is a smooth coefficient function, and e is white noise independent of X(⋅) with mean zero and variance $\sigma_{e}^{2}$.

The fundamental element in functional data analysis is the covariance function r(s,t). By Mercer’s theorem, r(s,t) can be written as $\sum_{l=1}^{\infty }\lambda_{\ell }\phi_{\ell }\left(s\right)\phi_{\ell }\left(t\right),$ where $\lambda_{1}\geq \lambda_{2}\geq \cdots \geq 0$ is the collection of eigenvalues and the $\phi_{\ell }\left(\cdot \right)$ are the associated eigenfunctions which satisfy $\int_{T}\phi_{\ell }\left(t\right)\phi_{\ell^{\prime }}\left(t\right)dt=1_{\left\{\ell =\ell^{\prime }\right\}}$. Here $1_{\left\{\cdot \right\}}$ is 1 if the condition inside the bracket holds and 0 otherwise. To ensure that β(t) is identifiable, we assume that the coefficient function β(t) can be written as $\sum_{\ell =1}^{K}\beta_{\ell }\phi_{\ell }\left(t\right),$ where the $\beta_{\ell }$ are scalars and, a possibly infinite K represents the number of non-zero eigenvalues.

### 2.2 Optimal design for predicting functions

Fix p≥1 and assume that p observations will be collected from a new subject. The goal is to select the p *optimal* sampling points in T for predicting the new subject’s curve with the smallest possible error.

Let $t={\left(t_{1},\ldots ,t_{p}\right)}^{\prime }\in T^{p}$ be the vector of sampling points and $W_{i^{*}}\left(t\right)={\left\{W_{i^{*}}\left(t_{1}\right),\ldots ,W_{i^{*}}\left(t_{p}\right)\right\}}^{\prime }$ be the noisy observations for a new subject $i^{*}$. Under model (1), the best predictor of $X_{i^{*}}\left(t\right)$ conditional on $W_{i^{*}}\left(t\right)$ is the best linear unbiased predictor (BLUP) of $X_{i^{*}}\left(t\right)$,

(3) $$\text{E}\left\{X_{i^{*}}\left(t\right)|W_{i^{*}}\left(t\right)\right\}=\mu \left(t\right)+r{\left(t,t\right)}^{\prime }\Sigma_{W}{\left(t\right)}^{-1}W_{i^{*}}\left(t\right),$$

where $r\left(t,t\right)={\left\{r\left(t,t_{1}\right),\ldots ,r\left(t,t_{p}\right)\right\}}^{\prime }\in R^{p}$ and $\Sigma_{W}\left(t\right)=\text{Cov}\left\{W_{i^{*}}\left(t\right)\right\}\in R^{p\times p}$. For simplicity, we suppress the notation t from $\Sigma_{W}\left(t\right)$ and use $\Sigma_{W}$. The optimal sampling points t can be selected by minimizing the mean integrated squared error of the BLUP,

(4) $$M_{1}\left(t\right)≔\text{E}\int_{T}{\left[X_{i^{*}}\left(t\right)-\text{E}\left\{X_{i^{*}}\left(t\right)|W_{i^{*}}\left(t\right)\right\}\right]}^{2}dt.$$

The optimal design is then defined as $t^{\text{opt}}≔\arg\min_{t\in T^{p}}M_{1}\left(t\right)$. And we simplify $M_{1}\left(t\right)$ as

$$M_{1}\left(t\right)=\text{E}\int {\left\{{\overset{¯}{X}}_{i^{*}}\left(t\right)\right\}}^{2}dt-\text{E}\int {\left[\text{E}\left\{{\overset{¯}{X}}_{i^{*}}\left(t\right)|W_{i^{*}}\left(t\right)\right\}\right]}^{2}dt=\int r\left(t,t\right)dt-\int r{\left(t,t\right)}^{\prime }\Sigma_{W}^{-1}r\left(t,t\right)dt=\int r\left(t,t\right)dt-\text{tr}\left(R\Sigma_{W}^{-1}\right),$$

where tr(⋅) is the trace operator and $R=\int r{\left(t,t\right)}^{\prime }r\left(t,t\right)dt\in R^{p\times p}$ whose $\left(j_{1},j_{2}\right)$ element is given by $\int r\left(t,t_{j_{1}}\right)r\left(t,t_{j_{2}}\right)dt=\sum_{\ell =1}^{K}\lambda_{\ell }^{2}\phi_{\ell }\left(t_{j_{1}}\right)\phi_{\ell }\left(t_{j_{2}}\right)$. We write $R=\Phi \left(t\right)\Lambda^{2}\Phi {\left(t\right)}^{\prime },$ where $\Phi \left(t\right)={\left[\phi_{\ell }\left(t_{j_{1}}\right)\right]}_{1\leq j_{1}\leq p,\;1\leq \ell \leq K}\in R^{p\times K}$ and $\Lambda =\text{diag}\left(\lambda_{1},\ldots ,\lambda_{K}\right)\in R^{K\times K}$. Note that $\int r\left(t,t\right)dt=\sum_{\ell =1}^{K}\lambda_{\ell }=\text{tr}\left(\Lambda \right).$ It is also easy to show that $\Sigma_{W}=\Phi \left(t\right)\Lambda \Phi {\left(t\right)}^{\prime }+\sigma_{\epsilon }^{2}I_{p}.$ Therefore,

$$M_{1}\left(t\right)=\text{tr}\left(\Lambda \right)-\text{tr}\left[\Phi \left(t\right)\Lambda^{2}\Phi {\left(t\right)}^{\prime }{\left\{\Phi \left(t\right)\Lambda \Phi {\left(t\right)}^{\prime }+\sigma_{\epsilon }^{2}I_{p}\right\}}^{-1}\right].$$

And if we let $S\left(t\right)=\Lambda \Phi {\left(t\right)}^{\prime }{\left\{\Phi \left(t\right)\Lambda \Phi {\left(t\right)}^{\prime }+\sigma_{\epsilon }^{2}I_{p}\right\}}^{-1}\Phi \left(t\right)\Lambda$, then we obtain the simplified form

(5) $$M_{1}\left(t\right)=\text{tr}\left(\Lambda \right)-\text{tr}\left\{S\left(t\right)\right\}.$$

### 2.3 Optimal design for predicting an outcome

Similar to Section 2.2, assume that p observations will be collected from a new subject indexed by $i^{*}$. But let the goal now be to select the p *optimal* sampling points in T for predicting the new subject’s scalar outcome with the smallest possible error.

Using the same notation as in Section 2.2, let $t={\left(t_{1},\ldots ,t_{p}\right)}^{\prime }\in T^{p}$ be the vector of sampling points and $W_{i^{*}}\left(t\right)={\left\{W_{i^{*}}\left(t_{1}\right),\ldots ,W_{i^{*}}\left(t_{p}\right)\right\}}^{\prime }$ be the noisy observations for subject $i^{*}$. Under the functional linear model (2), $\text{E}\left(Y_{i*}|X_{i^{*}}\right)=\alpha +\int_{T}{\overset{¯}{X}}_{i^{*}}\left(t\right)\beta \left(t\right)dt$, where ${\overset{¯}{X}}_{i^{*}}\left(t\right)=X_{i^{*}}\left(t\right)-\mu \left(t\right)$. Then under the functional data model (1), the best predictor of $Y_{i^{*}}$ conditional on $W_{i^{*}}\left(t\right)$ is the best linear unbiased predictor of $Y_{i^{*}}$,

$$\text{E}\left\{\text{E}\left(Y_{i^{*}}|X_{i^{*}}\right)|W_{i^{*}}\left(t\right)\right\}=\alpha +\int_{T}\text{E}\left\{{\overset{¯}{X}}_{i^{*}}\left(t\right)|W_{i^{*}}\left(t\right)\right\}\beta \left(t\right)dt.$$

And the mean squared error for predicting $\text{E}\left(Y_{i^{*}}|X_{i^{*}}\right)$ is

$$M_{2}\left(t\right)=\text{E}{\left[\int_{T}{\overset{¯}{X}}_{i^{*}}\left(t\right)\beta \left(t\right)dt-\int_{T}\text{E}\left\{{\overset{¯}{X}}_{i^{*}}\left(t\right)|W_{i^{*}}\left(t\right)\right\}\beta \left(t\right)dt\right]}^{2}.$$

Then the optimal design is $t^{\text{opt}}≔\arg\min_{t\in T^{p}}M_{2}\left(t\right).$ Note that the mean squared error for predicting $Y_{i^{*}}$ is $M_{2}\left(t\right)+\sigma_{e}^{2}$, which results in the same design. This design was studied in earlier work including Ritter (1996), and more recently Ji and Müller (2017).

By Eq. (3), $\int \text{E}\left\{{\overset{¯}{X}}_{i^{*}}\left(t\right)|W_{i^{*}}\left(t\right)\right\}\beta \left(t\right)dt={\left\{\int \beta \left(t\right)r\left(t,t\right)dt\right\}}^{\prime }\Sigma_{W}^{-1}W_{i^{*}}\left(t\right).$ Thus,

$$M_{2}\left(t\right)=\text{E}{\left\{\int {\overset{¯}{X}}_{i^{*}}\left(t\right)\beta \left(t\right)dt\right\}}^{2}-\text{E}{\left[{\left\{\int \beta \left(t\right)r\left(t,t\right)dt\right\}}^{\prime }\Sigma_{W}^{-1}W_{i^{*}}\left(t\right)\right]}^{2}=\iint r\left(s,t\right)\beta \left(s\right)\beta \left(t\right)dsdt-{\left\{\int \beta \left(t\right)r\left(t,t\right)dt\right\}}^{\prime }\Sigma_{W}^{-1}\left\{\int \beta \left(t\right)r\left(t,t\right)dt\right\}.$$

Then, $\iint r\left(s,t\right)\beta \left(s\right)\beta \left(t\right)dsdt=\sum_{\ell =1}^{K}\lambda_{\ell }\beta_{\ell }^{2}=\text{tr}\left(\beta \beta^{\prime }\Lambda \right),$ where β is a K-dimensional vector with $\beta ={\left(\beta_{1},\ldots ,\beta_{K}\right)}^{\prime }$. We also obtain $\int \beta \left(t\right)r\left(t,t_{j}\right)dt=\sum_{\ell =1}^{K}\lambda_{\ell }\beta_{\ell }\phi_{\ell }\left(t_{j}\right)$ which leads to ∫β(t)r(t,t)dt=Φ(t)Λβ. Therefore, we obtain the simplified form

(6) $$M_{2}\left(t\right)=\text{tr}\left(\beta \beta^{\prime }\Lambda \right)-\text{tr}\left\{\beta \beta^{\prime }S\left(t\right)\right\}.$$

### 2.4 A joint design for functional data

In practice, there might be multiple goals in design with each goal resulting in one optimal design. Depending on the goal, the corresponding optimal design may vary and may not be optimal for alternative goals. Indeed, the optimal sampling points for predicting functions may not be the optimal sampling points for predicting a scalar outcome, and vice versa. It may thus be useful to consider a joint design to balance between the different goals. Note that joint designs may also be referred to as compound designs in the statistical design literature ( Atkinson et al., 2007, Chapter 21).

Before formulating a joint design, consider first the design objective function

(7) $$M_{B}\left(t\right)=\text{tr}\left(B\Lambda \right)-\text{tr}\left\{BS\left(t\right)\right\},$$

where B is an arbitrary positive semidefinite matrix and we will call it a “linear design criterion matrix” as the objective function depends linearly on elements of B. The form in (7) is general with different B leading to different designs. In particular, it includes as special cases, the objective functions for the design for predicting functions (5) and for the design for predicting an outcome (6). Indeed, for predicting the growth curve of a new subject, B is the identity matrix and for predicting a scalar outcome of a new subject, $B=\beta \beta^{\prime }$. Additionally, if it is more important to predict a curve more accurately at some time points than others, one may consider a weighted mean integrated squared error $\text{E}\int_{T}w\left(t\right){\left[X_{i^{*}}\left(t\right)-\text{E}\left\{X_{i^{*}}\left(t\right)|W_{i^{*}}\left(t\right)\right\}\right]}^{2}dt$ for some known weight function 0≤w(t)≤1. It can be shown that the objective function to be minimized still takes the form in (7) with a particular design criterion matrix. Specifically, ${\left[B\right]}_{\ell ,\ell^{\prime }}=\int w\left(t\right)\phi_{\ell }\left(t\right)\phi_{\ell^{\prime }}\left(t\right)dt$ and is positive semidefinite with a finite operator norm by Lemma 1 in Appendix A.

Now consider a bivariate continuous function f(⋅,⋅) on [0,∞)×[0,∞) and the objective function $M\left(t\right)=f\left(M_{1}\left(t\right),\right)\left(M_{2}\left(t\right)\right)$. Suppose the joint design is to minimize the objective function M(t). It is reasonable to impose the following assumption on f(⋅,⋅):

Assumption 1 f(x,y) is nondecreasing along both x and y and f(0,0)=0. Moreover, $\lim_{x\to 0,y\to 0}f\left(x,y\right)=0$.

Let $w_{1}$ and $w_{2}$ be two fixed non-negative constants. Two sensible forms of f are: $f_{1}\left(x,y\right)=w_{1}x+w_{2}y$ and $f_{2}\left(x,y\right)=\max\left(w_{1}x,w_{2}y\right)$. The former is a joint design that minimizes a linear combination of two prediction errors while the latter means that the joint design aims to minimize the maximum of the two prediction errors (up to multiplicative weights). In particular, $f_{1}\left(M_{1}\left(t\right),M_{2}\left(t\right)\right)=M_{B}\left(t\right)$ with $B=w_{1}I+w_{2}\beta \beta^{\prime }$. It is straightforward to show that both forms satisfy Assumption 1. The two constants $w_{1}$ and $w_{2}$ are used to control the weights of the two different design objective functions. One reasonable choice of $w_{1}$ and $w_{2}$ is to balance the two design objective functions such that one design does not dominate the other. In view of (7) and Theorem 2 from the following section, we may let $w_{1}=1∕\text{tr}\left(\Lambda \right)$ and $w_{2}=1∕\text{tr}\left(\beta \beta^{\prime }\Lambda \right)$, and it can then be shown that $0\leq w_{1}M_{1}\left(t\right)\leq 1$ and $0\leq w_{2}M_{2}\left(t\right)\leq 1$.

## 3 Properties of M(t)

In this section we study the properties of M(t) for any function f(⋅,⋅) that satisfies Assumption 1. We assume that the random functions, X(t), are square integrable (i.e., $\text{E}\left\{\int_{T}X{\left(t\right)}^{2}dt\right\}<\infty$) and the coefficient function β(t) in the functional linear regression model (2) is also square integrable (i.e., $\int_{T}\beta^{2}\left(t\right)dt<\infty$). Proofs of the theorems are provided in Appendix A.

Theorem 1 *Suppose* $t\in {\left[0,1\right]}^{p}$*,* $\overset{˜}{t}\in {\left[0,1\right]}^{p+c}$ *for some fixed integer* c>0 *and* $t\subset \overset{˜}{t}$*, then* $M\left(\overset{˜}{t}\right)\leq M\left(t\right).$

Theorem 1 implies that more observations (i.e., larger p) do not increase the value of the objective function M(t).

We also study the deterministic bound of M(t) as p diverges to infinity according to a fixed design.

Theorem 2 *Suppose that the assumptions stated in* Appendix A *hold. For the fixed design where* $t_{p}={\left\{0,\;1∕p,\;2∕p,\ldots ,\;\left(p-1\right)∕p\right\}}^{\prime }$*, we have* $\lim_{p\to \infty }M\left(t_{p}\right)=0$*.*

Theorem 2 provides the rationale that a dense set of time points in T is sufficient as the candidate sampling points. In practice, because of the cost for data collection and other considerations, a small number of sampling points with reasonable prediction power might be preferred. In Section 4.2, we propose a data-driven method for selecting the number of optimal time points.

## 4 Implementation

### 4.1 Model estimation using pilot data

To implement the proposed optimal design, we need to estimate the covariance function r(s,t), error variance $\sigma_{\epsilon }^{2}$ and coefficient function β(t) using pilot data. Many methods exist for covariance function estimation including local polynomial regression (Yao et al., 2005), mixed effects models (James et al., 2000) and geometric PCA (Peng and Paul, 2009). We use the fast covariance estimation method (FACEs) from Xiao et al. (2017), which uses a penalized tensor product of cubic B-splines for approximating the true covariance function. The error variance $\sigma_{\epsilon }^{2}$ can also be estimated by FACEs. As for estimating β(t), we select K, the number of eigenfunctions, by the percentage of variance explained (PVE) with a value of 0.95.

### 4.2 Optimization algorithm and selection of number of design points

In practice, the optimal sampling points are selected from a pre-determined set of candidate time points, denoted by s. Theorem 2 suggests that equally spaced sampling points can form a reasonable set of candidate points. If the number of selected design points is small, then we use a full search algorithm (i.e., we evaluate M for every combination of p points from s). If the number of selected design points is large, a full search becomes computationally difficult and one may use a Monte Carlo sampling method in Wu et al. (2017) or a sequential search method in Ji and Müller (2017). In this paper we focus on the full search algorithm.

In many applications, the number of optimal time points p may not be known *a priori*. One approach is to choose the smallest p such that the expected error is smaller than some pre-determined tolerance error. Alternatively, similar to Ferraty et al. (2010), one may incorporate the cost of collecting more sampling points into consideration. Here we propose a new method for selecting p. First, when t=∅, an empty set, we define $M_{1}\left(\emptyset \right)=\text{tr}\left(\Lambda \right)$, $M_{2}\left(\emptyset \right)=\text{tr}\left(\beta \beta^{\prime }\Lambda \right)$, and $M\left(\emptyset \right)=f\left(M_{1}\left(\emptyset \right),M_{2}\left(\emptyset \right)\right)$. Note that $M_{1}\left(\emptyset \right)$ is the total variation of the functional predictor while $M_{2}\left(\emptyset \right)$ is the total variation of the response that can be explained by the functional predictor. Then it can be easily verified by the definitions in (5) and (6) and the assumptions on f that M(t)≤M(∅) for any t of any dimension. Let $t_{p}^{⋆}=\arg\min_{t\subseteq s,\;\;t\in T^{p}}M\left(t\right)$. Then,

(8) $$p^{*}≔\underset{p\in N}{\min}\left\{M\left(t_{p}^{⋆}\right)∕M\left(\emptyset \right)+\delta p\right\},$$

where 0<δ<1 is a fixed constant corresponding to the maximum percent reduction in expected squared error gained by augmenting the design with an additional design point.

By Theorem 1 in Section 3, for any p, $M\left(t_{p+1}^{⋆}\right)\leq M\left(t_{p}^{⋆}\right)\leq M\left(\emptyset \right)$. Thus, the relative error level $M\left(t_{p}^{⋆}\right)∕M\left(\emptyset \right)$ is a decreasing function of p and converges to 0 by Theorem 2. This implies that for any fixed δ>0, $p^{*}$ is finite. In practice, we plug estimated model components (see Section 4.1) into M to obtain M^. Then we let t^p⋆=argmint⊆s,t∈RpM^(t) and define

(9) p^∗≔minp∈NM^(t^p⋆)∕M^(∅)+δp.

Small values of δ seem preferable and we use δ=0.05 in both the data application and simulations. This implies that we select a p^ such that the addition of a new design point will result in no more than 5% of reduction in expected squared error with respect to the error reduced by using the fully observed functional predictor.

### 4.3 Software and shiny interactive graphic

The proposed optimal design method has been implemented as an R package (R Core Team, 2016) FDAdesign that includes interactive graphics using shiny (Chang et al., 2016) which can be used to evaluate design objectives corresponding to different sampling designs. The interface of the graphic is illustrated in the data application. Details about using the FDAdesign package and the interactive graphics can be found in Section S.1 of the Supplementary materials.

## 5 Application to fetal ultrasound

We apply the proposed methodology to fetal growth data, where ultrasound scans were performed at different weeks of gestational age (GA). For this analysis, we model measurements of abdominal circumference by ultrasound (scaled within the range of 0 and 1) to estimate individual fetal growth trajectories and use newborn birth weight as the scalar outcome.

**Fig. 1:**
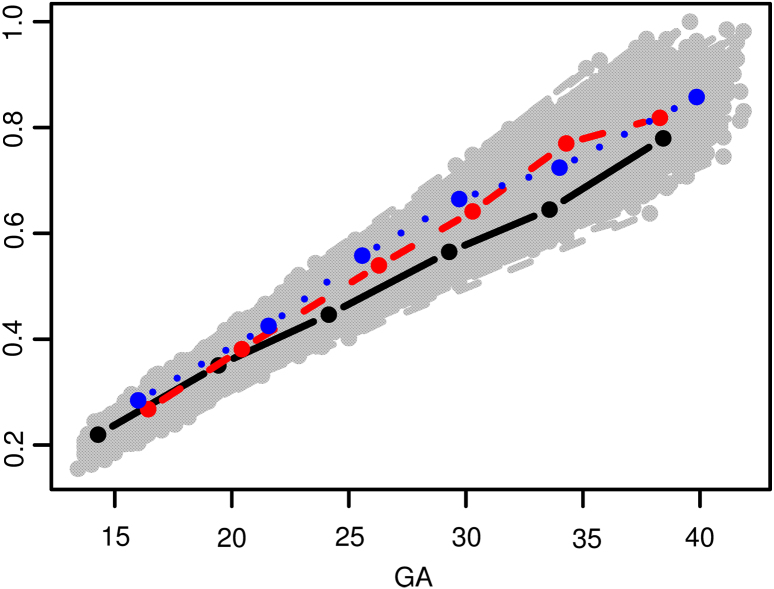
Spaghetti plot of the fetal ultrasound data.

The fetal growth dataset contains between 1 and 6 ultrasound scans for each of 2388 subjects, with most subjects having 5 scans. The spaghetti plot for the ultrasound measurements is shown in Fig. 1, with data from 3 subjects highlighted. While the 3 subjects do show some degree of curvilinearity, the overall pattern of trajectories raises the question of whether a linear mixed effects model would suffice for this data.

Thus, we compare the functional model and the linear mixed effects model using 10-fold cross validation. We find that the linear mixed effects model has twice the prediction error of the functional model. Figure S.2 in the Supplementary materials illustrates the prediction performance of the two models for one particular case, where 90% data are used for model estimation and the remaining 10% data are used for evaluation. Therefore, using functional model seems more appropriate for this application.

**Fig. 2:**
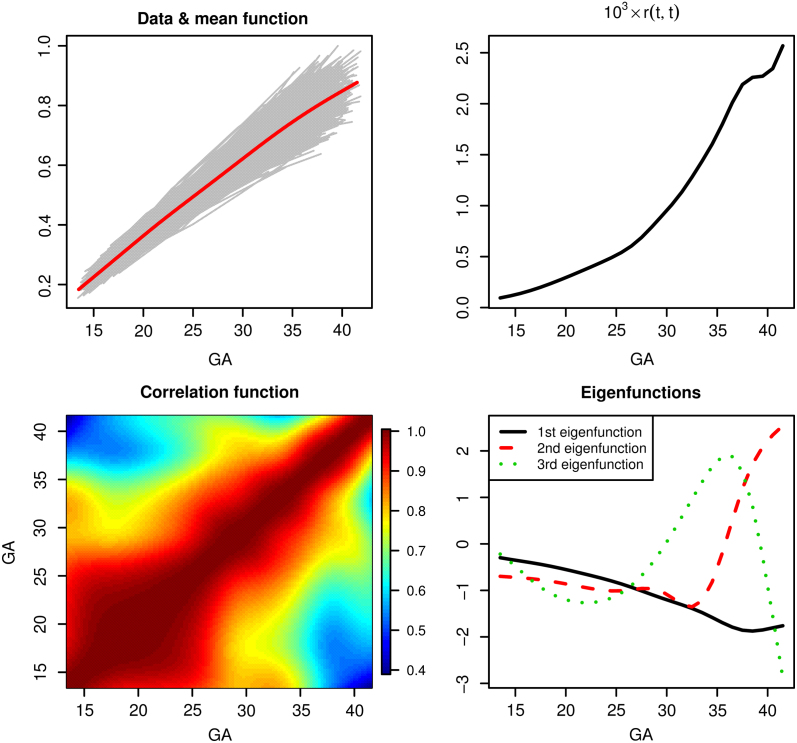
fPCA fit to the ultrasound measurements.

Fig. 2 displays the fPCA fit. The top panels of Fig. 2 show that both the estimated mean function and variance function are increasing with gestational age. The bottom left panel of Fig. 2 indicates high positive correlations (>0.8) when both gestational ages are smaller than 32 weeks. The top three estimated eigenvalues are $8.7\times 10^{-4}$, $1.0\times 10^{-4}$ and $0.3\times 10^{-5}$, respectively, with the corresponding estimated eigenfunctions shown in the bottom right panel of Fig. 2. The estimated error variance is $2.2\times 10^{-4}$.

When we predict subject birth weight using the functional linear model (2) with abdominal circumference as the functional covariate, it turns out that about 99% of the variation in the birth weight is explained by the functional covariate. For the estimated coefficient function; see Figure S.3 of the Supplementary materials.

Finally we consider a linear joint design with the target of accurately recovering the ultrasound measurements of fetal abdominal circumference and predicting the newborn outcome of birth weight. The objective function for the joint design is w1M^1(t)+w2M^2(t), where M^1(t) is the estimated objective function for recovering individual functions while M^2(t) is the estimated objective function for predicting a scalar outcome; see Section 2.4 for more details. To balance the two objective functions, we let $w_{1}={\left(\sum_{\ell =1}^{3}{\overset{ˆ}{\lambda }}_{\ell }\right)}^{-1}$ and $w_{2}={\left(\sum_{\ell =1}^{3}{\overset{ˆ}{\lambda }}_{\ell }{\overset{ˆ}{\beta }}_{\ell }^{2}\right)}^{-1}$, where the ${\overset{ˆ}{\lambda }}_{\ell }$ and the ${\overset{ˆ}{\beta }}_{\ell }$ are estimated from the fetal data and the top 3 eigenvalues are selected using a PVE of 0.95. The above weights ensure that 0≤w1M^1(t)≤1 and 0≤w2M^2(t)≤1. As a result, one objective function will not dominate the other.

**Fig. 3:**
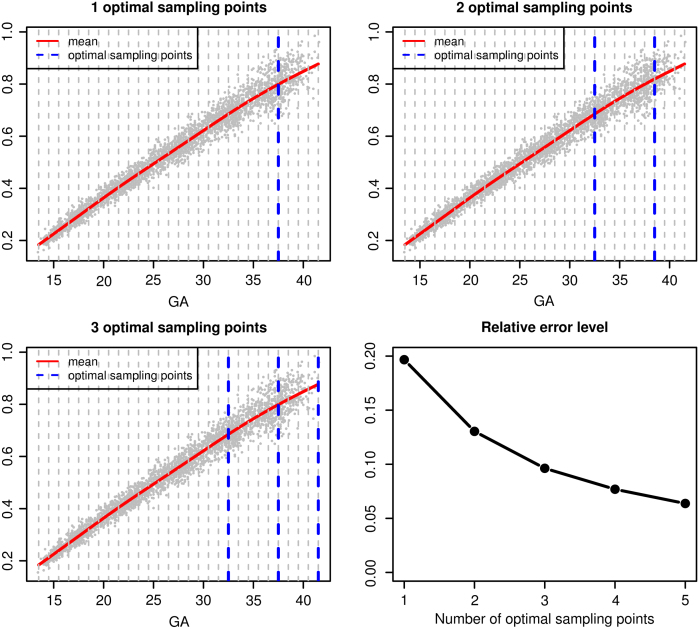
Optimal sampling points and the corresponding relative error levels. The dashed gray vertical lines in the top left, top right and bottom left panels are the candidate sampling points. The relative error levels are M^(tˆp)∕M^(∅), where ${\overset{ˆ}{t}}_{p}$ is the vector of p optimal sampling points determined by M^.

We let the set of candidate time points s be the collection of half weeks between 13 and 41 weeks gestational age. Using the proposed method, we determine the optimal sampling points when the number of sampling points p is fixed at 1, 2 and 3. We also calculate the relative error M^∕M^(∅) and Fig. 3 displays the results. The top left panel of Fig. 3 shows that if only 1 sampling point is selected, then 37 weeks is the optimal time point for collecting the ultrasound measurement and its relative error is about 0.20 (bottom right panel of Fig. 3). If 2 sampling points are desired, then 32 and 38 weeks are the optimal time points for collecting ultrasound measurements. With 2 optimal sampling points, the relative error is 0.13, which is smaller than the relative error with only 1 optimal sampling point. The bottom right panel of Fig. 3 displays the relative error with several values of p. As expected, the relative error decreases as p increases. Using the selection criterion (9) with δ=0.05, we determine that optimally, 2 sampling points would be selected.

**Fig. 4:**
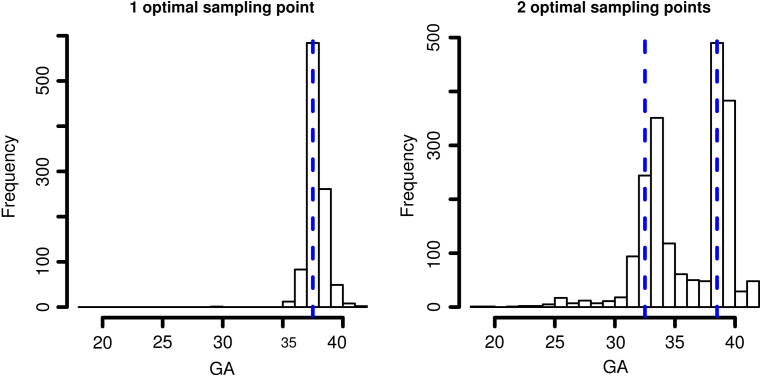
Histograms of selected optimal scan weeks from 1000 bootstrapped datasets for p=1 and p=2. The blue dashed lines are the estimated optimal scan weeks using the original fetal ultrasound data.

To evaluate the uncertainty in the estimated optimal sampling points, we bootstrap the fetal ultrasound data at the subject level and select the optimal sampling points for 1000 bootstrapped datasets. Fig. 4 gives the histograms of the selected optimal sampling points, which show small variability of the estimated optimal sampling points. For example, for p=1, week 37 is selected about 60% of the times.

Finally, we plot in Fig. 5 screenshots of the Shiny interface for the fetal ultrasound. The top panel displays the heat map of the objective function/prediction errors as a bivariate function of two scan weeks. The optimal weeks are highlighted. The heat map indicates that at least one sampling point needs to be no early than 33 weeks in order to obtain a relatively small prediction error. As these plots evaluate the prediction error of any combination of candidate sampling points, they can be used to find all candidate sampling points that give a prediction error smaller than certain fixed error. The interface is interactive as users can select the first scan weeks and then the application will find the optimal second scan weeks. Moreover, users can go further by selecting the second scan weeks and compare the results with the optimal scan weeks. For example, as illustrated in the bottom panel, 13 weeks is selected for the first scan, then the 37 weeks is found to be optimal second scan weeks (left plot in the bottom panel). If 16 weeks is also selected for the second scan, then the result can be compared with several different choices including the optimal scan weeks (right plot in the bottom panel). A similar screenshot with the goal of selecting just one scan is presented in Figure S.4 of the Supplementary materials.

Fig. 5Screenshots of the interface of the Shiny application for the fetal ultrasound.

**Fig. 5(a):**
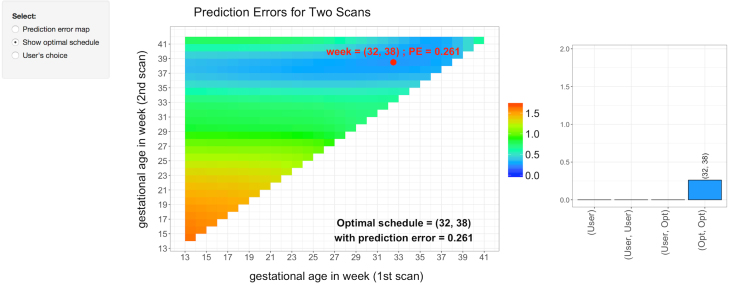
(a) Heat map of the objective function M^(t) evaluated with two scans.

**Fig. 5(b):**
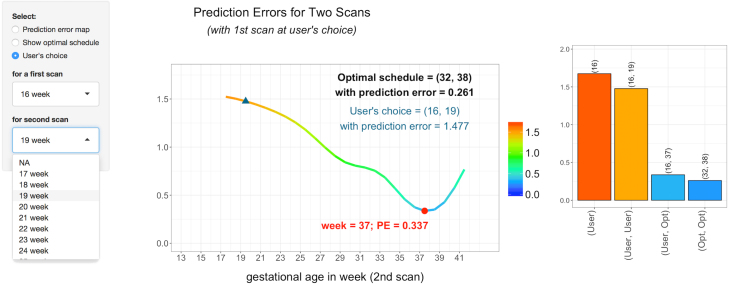
(b) Objective function M^(t) evaluated with two scans while one fixed at 16 weeks.

## 6 A simulation study

We conduct a simulation study to investigate the performance of the proposed design for (a) estimating optimal sampling points and for (b) selecting the number of optimal sampling points. We also compare functional data models against a parametric mixed effects model in terms of estimating optimal sampling points, when data is generated from either a functional data model or a parametric mixed effects model. We focus on the linear joint design where the goal is to best predict both an underlying true curve and a scalar outcome and we use the same design criterion matrix in the data example with weights $w_{1}={\left(\sum_{\ell }\lambda_{\ell }\right)}^{-1}$ and $w_{2}={\left(\sum_{\ell }\lambda_{\ell }\beta_{\ell }^{2}\right)}^{-1}$.

### 6.1 Simulation settings

For each simulation scenario, we use 200 Monte Carlo samples from the model in (1). For simplicity, we let the mean function μ(t) be zero for all t. We generate $X_{i}\left(t\right)$ by $X_{i}\left(t\right)=\sum_{\ell =1}^{5}\xi_{i\ell }\phi_{\ell }\left(t\right)$, where $\left\{\phi_{1}\left(t\right),\ldots ,\phi_{5}\left(t\right)\right\}$ is a set of orthonormal eigenfunctions (to be specified later) and $\xi_{i\ell }$ is sampled from a normal distribution with mean zero and variance $\lambda_{\ell }=10∕2^{\ell }$. Random errors $\epsilon_{ij}$ are sampled independently from a normal distribution with mean zero and variance $\sigma_{\epsilon }^{2}=9.6875$, which implies that the signal to noise ratio $\sigma_{\epsilon }^{-2}\sum_{\ell =1}^{5}\lambda_{\ell }$ equals one. The number of observations per subject varies across subjects and the sampling time points are drawn from the uniform distribution in the unit interval.

We consider a factorial design with three experimental factors:

E1. Covariance function r(s,t):
(a) *Periodic* covariance r(s,t) induced by five Fourier bases: $\phi_{\ell }\left(t\right)=\sqrt{2}\text{ sin}\left(\left(\ell +1\right)\pi t\right)$ for odd ℓ and $\phi_{\ell }\left(t\right)=\sqrt{2}\text{ cos}\left(\ell \pi t\right)$ for even ℓ. The covariance function is periodic because r(s,t)=r(1−s,t).
(b) *Non-periodic* covariance r(s,t) induced by five eigenfunctions shown in Figure S.6 of the Supplementary materials and the eigenfunctions do not have analytical forms.
E2. Number of observations per subject:
(a) $m_{i}∼\text{Uniform}\left\{3,4,5\right\}$ and (b) $m_{i}∼\text{Uniform}\left\{7,\ldots ,10\right\}$.
E3. Number of subjects: (a) n=400; (b) n=800; and (b) n=1500.

Thus, in total there are 12 model conditions to examine.

Then the scalar outcomes, Y, are generated from the functional linear model in (2). For simplicity, we let intercept α=0. We use four different coefficient functions β(t) (see Figure S.7 of the Supplementary materials):

FLM-Case1 $\beta \left(t\right)=\sum_{\ell =1}^{5}\beta_{\ell }\phi_{\ell }\left(t\right)$, with $\beta_{\ell }=4,2.5,1.5,1,$ and 0.5.
FLM-Case2 $\beta \left(t\right)=12t^{2}$.
FLM-Case3 $\beta \left(t\right)=4{\left(t-0.5\right)}^{2}+4\text{sin}\left(2\pi t\right)+2\text{cos}\left(6\pi t\right)$.

Note that in FLM-Case1 the coefficient function β(t) depends on the eigenfunctions $\phi_{\ell }\left(t\right)$ and is different for the periodic and non-periodic covariances (see Figure S.7 of the Supplementary materials). Random errors in (2) were sampled independently from a normal distribution with mean zero and variance $\sigma_{e}^{2}=4$.

### 6.2 Results for estimation of optimal sampling points

We consider estimation of optimal sampling points when the number of optimal points p is fixed at either 3, 4 or 5. Let $t_{p}^{*}$ be the p optimal sampling points that minimize the true objective function M(⋅) and let t^p be the p selected sampling points that minimize the estimated objective function M^(⋅). We evaluate the accuracy of the estimated optimal sampling points using the following evaluation criterion:

(10) AREp,isim=|M(tp∗)−Mt^p,isim|M(tp∗).

The absolute relative error, ${\mathrm{ARE}}_{p,i_{sim}}$, measures how close the expected (integrated) squared error using observations collected at the p estimated optimal sampling points is to the expected (integrated) squared error using the p true optimal points. We compare between $M\left(t_{p}^{*}\right)$ and Mt^p,isim, rather than between t^p and $t_{p}^{*}$, for the following reasons. First, when the covariance function r(s,t) is periodic as the one shown in the top left panel of Figure S.6 of the Supplementary materials, $t_{p}^{*}$ is not identifiable. This is because with a periodic covariance function, data (excluding random errors) collected at any sampling point in the left half of the domain is the same as data collected at one sampling point in the right half. The identifiability issue is illustrated in Section S.3.2 of the Supplementary materials. Second, as our ultimate goal is to minimize M(⋅), the expected (integrated) squared error, we consider that the measure ${\text{ARE}}_{p,i_{sim}}$ is more appropriate.

In additional to functional data methods, we consider the following linear mixed effects (LME) model, $W_{ij}=b_{i0}+b_{i1}t_{ij}+\epsilon_{ij},$ for estimating the covariance function r(s,t), where $b_{i0}$ and $b_{i1}$ are subject-specific random intercept and slope, respectively. The above model leads to a quadratic covariance function. In the following tables we use the labels, *non-parametric* and *parametric*, to indicate covariance estimation using the functional data model and using the linear mixed effects model, respectively.

The results with the periodic covariance function are summarized in Table 1. The proposed design works well and the ARE decreases as a function of number of subjects n and number of observations per subject $m_{i}$. The improved performance is due to improved estimation accuracy of the covariance function (and associated eigenfunctions and eigenvalues) as well as of the error variance of the random errors (results not shown). The results with the non-periodic covariance function are similar and are shown in Section S.3.3 of the Supplementary materials.

In addition to the ARE measure, we study the behavior of the objective function M for different choices of p by investigating the median and interquartile range (IQR) of the Mt^p,isim. The statistics for the periodic and non-periodic covariance cases are presented in Tables S.1 and S.2 of the Supplementary materials, respectively. The true objective function M(⋅) depends on the true covariance function r(s,t), n, $m_{i}$, and β(t). Thus Mt^p,isim can only be compared across different p, but not across different simulation settings with different n or $m_{i}$.

As expected Mt^p decreases with more number of optimal sampling points. The same holds when we use the *parametric* covariance estimation, estimating r(s,t) using the LME model. Because the LME model is misspecified for modeling functional data, selecting more optimal points by the *parametric* estimation has only a slight effect on improving the prediction accuracy. In all cases the proposed method with the *non-parametric* covariance estimation gives a smaller prediction error than the *parametric* estimation. When data are generated from the LME model, the proposed method performs equally well with both the *non-parametric* and *parametric* covariance estimation; see Section S.3.4 of the Supplementary materials. In conclusion, the proposed method with *non-parametric* covariance estimation using the fPCA model performs well on data with both simple and complex covariance structures.

**Table 1:** Median of absolute relative errors, $\left\{{\text{ARE}}_{p,i_{sim}}:i_{sim}=1,\ldots ,200\right\}$ and the corresponding interquartile ranges (IQR) in parentheses for the case of the periodic covariance.

			**Joint-Case1**		**Joint-Case2**		**Joint-Case3**	
			*Non-parametric*	*Parametric*	*Non-parametric*	*Parametric*	*Non-parametric*	*Parametric*
p=3								

	n=400	$m_{i}∼\left\{3,4,5\right\}$	0.019 (0.019)	1.292 (0.032)	0.018 (0.033)	1.507 (0.284)	0.055 (0.053)	1.598 (0.006)
		$m_{i}∼\left\{7,\ldots ,10\right\}$	0.011 (0.016)	1.537 (0.264)	0.010 (0.015)	1.507 (0.284)	0.024 (0.026)	1.598 (0.006)
	n=800	$m_{i}∼\left\{3,4,5\right\}$	0.011 (0.016)	1.537 (0.000)	0.010 (0.023)	1.507 (0.000)	0.030 (0.037)	1.598 (0.006)
		$m_{i}∼\left\{7,\ldots ,10\right\}$	0.005 (0.011)	1.537 (0.264)	0.009 (0.007)	1.507 (0.284)	0.012 (0.014)	1.598 (0.006)
	n=1500	$m_{i}∼\left\{3,4,5\right\}$	0.011 (0.014)	1.537 (0.000)	0.010 (0.014)	1.507 (0.000)	0.026 (0.024)	1.601 (0.006)
		$m_{i}∼\left\{7,\ldots ,10\right\}$	0.005 (0.007)	1.537 (0.264)	0.005 (0.007)	1.507 (0.284)	0.012 (0.010)	1.598 (0.006)

p=4								

	n=400	$m_{i}∼\left\{3,4,5\right\}$	0.047 (0.030)	1.585 (0.175)	0.046 (0.037)	1.612 (0.286)	0.070 (0.054)	1.983 (0.099)
		$m_{i}∼\left\{7,\ldots ,10\right\}$	0.015 (0.023)	1.676 (0.000)	0.016 (0.023)	1.612 (0.000)	0.027 (0.035)	1.983 (0.000)
	n=800	$m_{i}∼\left\{3,4,5\right\}$	0.031 (0.040)	1.676 (0.264)	0.029 (0.043)	1.612 (0.286)	0.045 (0.041)	1.983 (0.025)
		$m_{i}∼\left\{7,\ldots ,10\right\}$	0.012 (0.013)	1.676 (0.000)	0.011 (0.011)	1.612 (0.000)	0.02 (0.022)	1.983 (0.000)
	n=1500	$m_{i}∼\left\{3,4,5\right\}$	0.018 (0.024)	1.676 (0.000)	0.018 (0.026)	1.612 (0.000)	0.033 (0.032)	1.983 (0.000)
		$m_{i}∼\left\{7,\ldots ,10\right\}$	0.007 (0.010)	1.676 (0.000)	0.007 (0.010)	1.612 (0.000)	0.008 (0.016)	1.983 (0.000)

p=5								

	n=400	$m_{i}∼\left\{3,4,5\right\}$	0.059 (0.051)	1.713 (0.050)	0.051 (0.044)	1.929 (0.342)	0.063 (0.070)	2.167 (0.020)
		$m_{i}∼\left\{7,\ldots ,10\right\}$	0.027 (0.027)	1.695 (0.321)	0.022 (0.027)	1.587 (0.342)	0.026 (0.028)	2.167 (0.020)
	n=800	$m_{i}∼\left\{3,4,5\right\}$	0.039 (0.037)	2.016 (0.321)	0.039 (0.037)	1.929 (0.342)	0.043 (0.041)	2.167 (0.020)
		$m_{i}∼\left\{7,\ldots ,10\right\}$	0.020 (0.022)	1.695 (0.321)	0.013 (0.020)	1.587 (0.342)	0.016 (0.016)	2.167 (0.020)
	n=1500	$m_{i}∼\left\{3,4,5\right\}$	0.029 (0.027)	2.016 (0.321)	0.029 (0.031)	1.929 (0.342)	0.032 (0.036)	2.167 (0.020)
		$m_{i}∼\left\{7,\ldots ,10\right\}$	0.009 (0.017)	1.695 (0.000)	0.007 (0.009)	1.587 (0.000)	0.010 (0.015)	2.167 (0.020)

Note: **Joint-Case1** indicates that the scalar responses are generated using β(t) in FLM-Case1; similarly, **Joint-Case2** corresponds to FLM-Case2 and **Joint-Case3** to FLM-Case3. *non-parametric* and *Parametric* refer to the covariance estimation using the fPCA and LME models, respectively.

### 6.3 Results for selection of number of optimal sampling points

Now we evaluate the performance of the proposed method in (9) for selecting the number of optimal sampling points p. We use δ=0.05 and the true number of optimal points $p^{*}$ determined by (8) is 3. The performance of the proposed method is assessed in terms of the proportion of selecting the correct number of optimal sampling points, Nsim−1∑isim=1Nsim1p^isim∗=3, where $1_{\left\{\cdot \right\}}$ is an indicator function and p^isim∗ is the number of optimal sampling points determined by (9) using the ith simulated data.

The simulation results are presented in Table 2. We see that the performance of the proposed method is excellent for all cases. The results for the non-periodic function are similarly good and presented in Table S.4 of the Supplementary materials.

**Table 2:** Proportion of selected number of points being equal to 3 for the case of the periodic covariance.

		**Joint-Case1**	**Joint-Case2**	**Joint-Case3**
n=400	$m_{i}∼\left\{3,4,5\right\}$	0.94	0.97	0.94
	$m_{i}∼\left\{7,\ldots ,10\right\}$	0.98	0.99	0.95
n=800	$m_{i}∼\left\{3,4,5\right\}$	0.98	0.99	0.95
	$m_{i}∼\left\{7,\ldots ,10\right\}$	0.99	1.00	0.98
n=1500	$m_{i}∼\left\{3,4,5\right\}$	0.99	0.99	0.96
	$m_{i}∼\left\{7,\ldots ,10\right\}$	1.00	1.00	0.99

Note: **Joint-Case1** indicates that the scalar responses are generated using β(t) in FLM-Case1; similarly, **Joint-Case2** corresponds to FLM-Case2 and **Joint-Case3** to FLM-Case3.

### 6.4 Uncertainty of estimated optimal sampling points

To assess the uncertainty of the optimal sampling points estimated from the proposed method, we use a bootstrap approach as in the data application. For each simulated data, we bootstrap at the subject level, select optimal sampling points from the model estimation based on the bootstrapped data, and calculate the third quartile and 90% percentile of absolute relative errors in (10). The medians of the percentiles are presented in Tables S.7 and S.8 of the Supplementary materials. The results show good stability of the estimated optimal sampling points, which gets better when either the sample size or the number of observations per subject increases.

## Appendix A Assumptions and proofs

Assumptions for Theorem 2 Similar to Bunea and Xiao (2015), we make the following assumptions on the covariance function r(s,t): *Assumption A.* r(s,t) is continuous and a positive semi-definite kernel. *Assumption B.* $\sup_{k}\sup_{s\in T}\left|\phi_{k}\left(s\right)\right|$ is bounded by a constant. *Assumption C.* $\frac{\partial r\left(t,t\right)}{\partial t}$ is continuous in T. Moreover, $\int \left|\frac{\partial r\left(t,t\right)}{\partial t}\right|dt$ is finite. *Assumption D.* For all k, $\sup_{s\in T}\left|\phi_{k}^{\left(1\right)}\left(s\right)\right|\leq Ck^{\gamma_{1}}$, where $\phi_{k}^{\left(1\right)}\left(s\right)$ is the derivative of $\phi_{k}$ and $C,\gamma_{1}$ are positive constants. *Assumption E.* There exist constants $C_{1}$, $C_{2}$ and $\beta_{2}\geq \beta_{1}>\max\left(\gamma_{1},1\right)$ such that $C_{2}k^{-\beta_{2}}\leq \lambda_{k}\leq C_{1}k^{-\beta_{1}}$, for all k. Moreover, there exist constants $C_{3}$ and $\beta_{3}>\beta_{2}$ such that $\lambda_{k}-\lambda_{k+1}\geq C_{3}k^{-\beta_{3}}$, for all k.

*Finally, we assume that the linear design matrix* B *is positive semidefinite with a finite operator norm.*

Lemma 1 *If* 0≤w(t)≤1 *for* t∈T *for a bounded interval* T*. Then* $B={\left[b_{\ell ,\ell^{\prime }}\right]}_{1\leq \ell ,\ell^{\prime }\leq \infty }$ *with* $b_{\ell ,\ell^{\prime }}=\int w\left(t\right)\phi_{\ell }\left(t\right)\phi_{\ell^{\prime }}\left(t\right)dt$ *is positive semidefinite with a finite operator norm.*

Proof It suffices to prove that $x^{\prime }Bx$ is non-negative and finite for any $x={\left(x_{1},x_{2}\ldots ,x_{K}\right)}^{\prime }$ with $\sum_{\ell }x_{\ell }^{2}=1$. Indeed,

$$x^{\prime }Bx=\underset{\ell }{\sum }\underset{\ell^{\prime }}{\sum }x_{\ell }x_{\ell^{\prime }}\int w\left(t\right)\phi_{\ell }\left(t\right)\phi_{\ell^{\prime }}\left(t\right)dt=\int w\left(t\right)\underset{\ell }{\sum }\underset{\ell^{\prime }}{\sum }x_{\ell }x_{\ell^{\prime }}\phi_{\ell }\left(t\right)\phi_{\ell^{\prime }}\left(t\right)dt=\int w\left(t\right){\left\{\underset{\ell }{\sum }x_{\ell }\phi_{\ell }\left(t\right)\right\}}^{2}dt\geq 0,$$

and furthermore

$$x^{\prime }Bx=\int w\left(t\right){\left\{\underset{\ell }{\sum }x_{\ell }\phi_{\ell }\left(t\right)\right\}}^{2}dt\leq \int {\left\{\underset{\ell }{\sum }x_{\ell }\phi_{\ell }\left(t\right)\right\}}^{2}dt\leq \underset{\ell }{\sum }\underset{\ell^{\prime }}{\sum }x_{\ell }x_{\ell^{\prime }}\int \phi_{\ell }\left(t\right)\phi_{\ell^{\prime }}\left(t\right)dt\leq \underset{\ell }{\sum }x_{\ell }^{2}=1.\;□$$

Proof of Theorem 1 In light of Assumption 1 and Eq. (7), it suffices to prove the theorem for the design objective function in (7) with an arbitrary positive semidefinite matrix B. Define the symmetric matrix F(t) as in Lemma 2, then $F\left(\overset{˜}{t}\right)-F\left(t\right)$ is positive semidefinite and $S\left(t\right)=\Lambda^{1∕2}F\left(t\right)\Lambda^{1∕2}$ and $S\left(\overset{˜}{t}\right)=\Lambda^{1∕2}F\left(\overset{˜}{t}\right)\Lambda^{1∕2}$. For simplicity we denote F(t) by F and $F\left(\overset{˜}{t}\right)$ by $\overset{˜}{F}$. Since $\overset{˜}{F}-F$ is positive semidefinite, there exists a matrix ${\left(\overset{˜}{F}-F\right)}^{1∕2}$ such that $\overset{˜}{F}-F={\left(\overset{˜}{F}-F\right)}^{1∕2}{\left(\overset{˜}{F}-F\right)}^{1∕2}$. Then

$$M\left(\overset{˜}{t}\right)-M\left(t\right)=\text{tr}\left(B\Lambda^{1∕2}\overset{˜}{F}\Lambda^{1∕2}-B\Lambda^{1∕2}F\Lambda^{1∕2}\right)=\text{tr}\left\{B\Lambda^{1∕2}\left(\overset{˜}{F}-F\right)\Lambda^{1∕2}\right\}=\|{\left(\overset{˜}{F}-F\right)}^{1∕2}\Lambda^{1∕2}B^{1∕2}{\|}_{F}^{2}\geq 0.$$

In the above, $\|\cdot {\|}_{F}$ is the Frobenius norm of a matrix.  □

Lemma 2 *Suppose* $t\in T^{p}$*,* $\overset{˜}{t}\in T^{p+c}$ *for some fixed integer* c>0 *and* T *is a bounded interval. Define* $F\left(t\right)=\Lambda^{1∕2}\Phi {\left(t\right)}^{\prime }{\left\{\Phi \left(t\right)\Lambda \Phi {\left(t\right)}^{\prime }+\sigma_{\epsilon }^{2}I_{p}\right\}}^{-1}\Phi \left(t\right)\Lambda^{1∕2}$*. Then if* $t\subseteq \overset{˜}{t}$*,* $F\left(\overset{˜}{t}\right)-F\left(t\right)$ *is positive semidefinite.*

Proof Without of loss of generality we assume that $\sigma_{\epsilon }^{2}=1$. Let $A=\Phi \left(\overset{˜}{t}\right)\Lambda^{1∕2}$ and $A_{1}=\Phi \left(t\right)\Lambda^{1∕2}$. Then A can be partitioned as $A={\left(A_{1}^{\prime },A_{2}^{\prime }\right)}^{\prime }$ (after some proper permutation of the index in $\overset{˜}{t}$), where $A_{1}$ and $A_{2}$ are of dimensions p×K and c×K, respectively. It follows that

$$AA^{\prime }+I_{p}=\left(\begin{array}{cc} A_{1}A_{1}^{\prime }+I_{p} & A_{1}A_{2}^{\prime }\\ A_{2}A_{1}^{\prime } & A_{2}A_{2}^{\prime }+I_{c} \end{array}\right).$$

Now let $A_{11}=A_{1}A_{1}^{\prime }+I_{p}$, $A_{12}=A_{21}^{\prime }=A_{1}A_{2}^{\prime }$ and $A_{22}=A_{2}A_{2}^{\prime }+I_{c}$. Then

$${\left(AA^{\prime }+I_{p}\right)}^{-1}=\left(\begin{array}{cc} A_{11}^{-1}+A_{11}^{-1}A_{11}^{-1}A_{12}A_{22\cdot 1}^{-1}A_{21}A_{11}^{-1} & -A_{11}^{-1}A_{12}A_{22\cdot 1}^{-1}\\ -A_{22\cdot 1}^{-1}A_{12}A_{11}^{-1} & A_{22\cdot 1}^{-1} \end{array}\right),$$

where $A_{22\cdot 1}=A_{22}-A_{21}A_{11}^{-1}A_{12}$. Note that (i) $F\left(t\right)=A_{1}^{\prime }A_{11}^{-1}A_{1}$ and

$$\text{(ii) }F\left(\overset{˜}{t}\right)=A^{\prime }{\left(AA^{\prime }+I_{p}\right)}^{-1}A=\left(\begin{array}{cc} A_{1}^{\prime }, & A_{2}^{\prime } \end{array}\right){\left(AA^{\prime }+I_{p}\right)}^{-1}{\left(\begin{array}{cc} A_{1}^{\prime }, & A_{2}^{\prime } \end{array}\right)}^{\prime }$$

$$=A_{1}^{\prime }\left(A_{11}^{-1}+A_{11}^{-1}A_{12}A_{22\cdot 1}^{-1}A_{21}A_{11}^{-1}\right)A_{1}-A_{1}^{\prime }A_{11}^{-1}A_{12}A_{22\cdot 1}^{-1}A_{2}-\;\;\;\;\;\;\;{\left(A_{1}^{\prime }A_{11}^{-1}A_{12}A_{22\cdot 1}^{-1}A_{2}\right)}^{\prime }+A_{2}^{\prime }A_{22\cdot 1}^{-1}A_{2}.$$

It follows that

$$F\left(\overset{˜}{t}\right)-F\left(t\right)=A_{1}^{\prime }A_{11}^{-1}A_{12}A_{22\cdot 1}^{-1}A_{21}A_{11}^{-1}A_{1}-A_{1}^{\prime }A_{11}^{-1}A_{12}A_{22\cdot 1}^{-1}A_{2}-\;\;\;\;\;\;{\left(A_{1}^{\prime }A_{11}^{-1}A_{12}A_{22\cdot 1}^{-1}A_{2}\right)}^{\prime }+A_{2}^{\prime }A_{22\cdot 1}^{-1}A_{2}=B_{1}^{\prime }B_{1}-B_{1}^{\prime }B_{2}-B_{2}^{\prime }B_{1}+B_{2}B_{2}^{\prime }={\left(B_{1}-B_{2}\right)}^{\prime }\left(B_{1}-B_{2}\right),$$

where $B_{1}=A_{22\cdot 1}^{-1∕2}A_{21}A_{11}^{-1}A_{1}$ and $B_{2}=A_{22\cdot 1}^{-1∕2}A_{2}$. Therefore, we derive that

$$F\left(\overset{˜}{t}\right)-F\left(t\right)={\left(B_{1}-B_{2}\right)}^{\prime }\left(B_{1}-B_{2}\right),$$

which is always positive semidefinite.  □

Proof of Theorem 2 In light of Assumption 1 and Eq. (7), it suffices to prove the theorem for the design objective function in (7) with an arbitrary positive semidefinite matrix B of finite operator norm. We first focus on the fixed design. Without of loss of generality we assume that $\sigma_{\epsilon }^{2}=1$. Let $UDU^{\prime }$ be the eigendecomposition of $\Phi \left(t_{p}\right)\Lambda \Phi {\left(t_{p}\right)}^{\prime }$. Let $V=U^{\prime }\Phi \left(t_{p}\right)\Lambda^{1∕2}$. Note that we drop the dependence of U and V on p for simplicity. We also simply denote $\Phi \left(t_{p}\right)$ by Φ. Then $VV^{\prime }=D$ and $\Phi \left(t_{p}\right)\Lambda^{1∕2}=UV$. It follows that

$$\Lambda^{1∕2}\Phi^{\prime }\Sigma^{-1}\Phi \Lambda^{1∕2}={\left(UV\right)}^{\prime }{\left(UDU^{\prime }+I_{p}\right)}^{-1}\left(UV\right)=V^{\prime }{\left(D+I\right)}^{-1}V.$$

Then we derive that

$$M\left(t\right)=\text{tr}\left[\Lambda^{1∕2}B\Lambda^{1∕2}\left\{I-V^{\prime }{\left(D+I\right)}^{-1}V\right\}\right]\leq \|B{\|}_{op}\text{tr}\left[\Lambda \left\{I-V^{\prime }{\left(D+I\right)}^{-1}V\right\}\right],$$

where $\|B{\|}_{op}$ denotes the operator norm of B. Therefore, it suffices to prove that as p→∞, $\text{tr}\left[\Lambda \left\{I-V^{\prime }{\left(D+I\right)}^{-1}V\right\}\right]\to 0$. Denote the jth diagonal of D by $d_{j}$. Let $V=\left(v_{jk}\right)$ and $U=\left[u_{1},\ldots ,u_{p}\right]$. Since $V=U^{\prime }\Phi \left(t_{p}\right)\Lambda^{1∕2}$, we obtain $v_{jk}=u_{j}^{\prime }\phi_{k}\lambda_{k}^{1∕2}$, where $\phi_{k}={\left\{\phi_{k}\left(t_{1}\right),\phi_{k}\left(t_{2}\right),\ldots ,\phi_{k}\left(t_{p}\right)\right\}}^{\prime }$. Fix an arbitrary small ϵ with 0<ϵ<1∕2. Then there exists an integer $K_{0}$ such that $\sum_{k>K_{0}}\lambda_{k}<\epsilon$. By Proposition 5.1 in Bunea and Xiao (2015), for any sufficiently large p (depending on ϵ), $\left|v_{kk}^{2}∕\left(p\lambda_{k}\right)-1\right|\leq \epsilon ,\left|d_{k}-p\lambda_{k}\right|\leq \epsilon \text{ for all }k\leq K_{0}.$ It follows that

$$\text{tr}\left[\Lambda \left\{I-V^{\prime }{\left(D+I\right)}^{-1}V\right\}\right]=\underset{k\geq 1}{\sum }\lambda_{k}\left(1-\underset{j\geq 1}{\sum }\frac{v_{jk}^{2}}{d_{j}+1}\right)$$

$$\leq \underset{1\leq k\leq K_{0}}{\sum }\lambda_{k}\left(1-\frac{v_{kk}^{2}}{p\lambda_{k}}\frac{p\lambda_{k}}{d_{k}+1}\right)+\underset{k>K_{0}}{\sum }\lambda_{k}$$

$$\leq \underset{1\leq k\leq K_{0}}{\sum }\lambda_{k}\left\{1-\frac{p\lambda_{k}}{p\lambda_{k}+\epsilon +1}\left(1-\epsilon \right)\right\}+\epsilon$$

$$\leq \underset{1\leq k\leq K_{0}}{\sum }\lambda_{k}\left(\frac{1-\epsilon^{2}}{p\lambda_{k}+\epsilon +1}+\epsilon \right)+\epsilon$$

$$\leq \epsilon \underset{1\leq k\leq K_{0}}{\sum }\lambda_{k}+\underset{1\leq k\leq K_{0}}{\sum }\frac{\lambda_{k}}{p\lambda_{k}+1+\epsilon }+\epsilon$$

$$\leq \epsilon \underset{1\leq k\leq K_{0}}{\sum }\lambda_{k}+K_{0}∕p+\epsilon .$$

Therefore, with a sufficient large p, we can make $\text{tr}\left[\Lambda \left\{I-V^{\prime }{\left(D+I\right)}^{-1}V\right\}\right]$ smaller than ϵ, up to a finite multiplicative constant. We have proved that $\lim_{p\to \infty }M\left(t_{p}\right)=0$ for the fixed design. For the random design, the key is to establish that

$$\left|v_{kk}^{2}∕\left(p\lambda_{k}\right)-1\right|=o_{P}\left(1\right),\;\;\left|d_{k}-p\lambda_{k}\right|=o_{P}\left(1\right)\text{ for all }k\leq K_{0},$$

which holds by a proposition (similar to Proposition 5.1 in Bunea and Xiao (2015)) in the Supplementary materials.  □

## Appendix B Supplementary data

MMC S1 Additional simulations and data application and software.
