# Supplementary material for: A joint design for functional data with application to scheduling ultrasound scans
Source: Comput Stat Data Anal. 2018 Jun;122:101–14. doi: 10.1016/j.csda.2018.01.009 (PMC5840761; doi:10.1016/j.csda.2018.01.009)
Supplement: MMC S1 — Additional simulations and data application and software. [file mmc1.pdf]

Supplementary Material for "A Joint Design for Functional Data with Application to Scheduling Ultrasound Scans"

So Young Park, Luo Xiao, Jayson D. Wilbur, Ana-Maria Staicu and N. L'ntshotsholé Jumbe

Section S.1 provides an overview of the `FDAdesign` package and interactive graphics for visualization. Section S.2 provides additional figures for the fetal ultrasound data application and application to the Berkeley growth data. Section S.3 presents additional simulation results.

Lastly, interactive graphics of the optimal design results for one simulated dataset are available at the following links: `https://park-ncsu-stat.shinyapps.io/JointOptDesign-demo/`.

S.1 R package `FDAdesign` with interactive graphics

The proposed method is implemented in `R` package `FDAdesign` which is available at `GitHub` and will be distributed on `CRAN` soon. The current version of the package can be installed by the following code:

```
> library(devtools)
> devtools::install_github('soyoung-park/FDAdesign')
```

The `FDAdesign` package includes three functions: `opt_design_fda()`, `selection_p()`, and `interactive_plot()`. The `opt_design_fda()` function implements the proposed method of selecting an optimal design for fixed p. For example, the code for finding an optimal sampling points for $p = 2$:

```
> opt_design_fda(p = 2, Phi, lambda, B , sigma2)
```

where `Phi`, `lambda`, `B`, and `sigma2` are eigenfunctions, eigenvalues, a design criterion matrix, and error variance, respectively. The function uses the grid of evaluation points for eigenfunctions as candidate points.

The `selection_p()` function implements the proposed method for determining number of optimal sampling points, p, given selection parameter, δ, defined in Section 4.2. The code for selecting p:

```
> selection_p(delta = 0.1, Phi, lambda, B, sigma2)
```

This function also returns the outputs of `opt_design_fda`() function as well as the relative error levels $\widehat{\mathcal{M}}(\mathbf{t}_p^*)/\widehat{\mathcal{M}}(\mathbf{s})$ for all $p = 1, \ldots, (\widehat{p}+1)$, where $\widehat{p}$ is the selected number of optimal points. It also provides a plot of $\widehat{\mathcal{M}}(\mathbf{t}_p^*)/\widehat{\mathcal{M}}(\varnothing) + \delta p$ with the given δ; see Figure S.1.

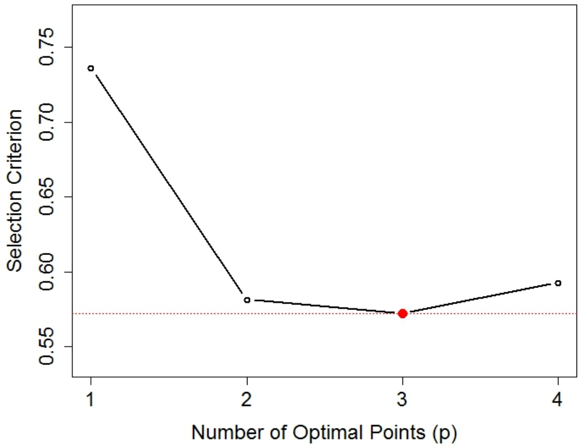

Figure S.1: Example of an output plot of the `selection_p()` function

Lastly, the `interactive_plot()` function returns a user interface with three tabs to help visualize and explore the estimated objective function $\widehat{\mathcal{M}}$ for $p = 1, 2, 3$. The code for producing graphics:

```
> opt.result <- selection_p(delta = 0.1, Phi, lambda, B, sigma2)
> interactive_plot(opt.result)
```

For illustration, we include a few screenshots of the interactive graphics made for the optimal design results of the fetal growth data; see Figure 4 in Section 5 and Figure S.4 in Section S.2.

The first tab shows a plot of the objective function (or, prediction error) evaluated at candidate points for the case of $p = 1$; a user can choose to indicate the optimal point on the plot and also can select arbitrary sampling point to compare prediction errors; see Figure S.4 for example. Similarly, the second tab shows the objective function evaluated at all possible pairs of the candidate points, with and without the optimal sampling points; see Figure 4(a). The user can also select an arbitrary time for the first sampling point or both the first and second points. When the first sampling point is selected by the user, the graphic produces a plot of the objective function for the second sampling point given the first one selected by the user; see Figure 4(b). On the plot, red point corresponds the optimal point for the second sample given the first one selected by the user, whereas blue triangular point corresponds two sampling points selected by the user. The third tab shows similar interactive graphics for the case of $p = 3$. For easy comparison of prediction errors, each tab includes a bar plot of prediction errors corresponding to the user-selected points and the optimal sampling points.

S.2 Data Applications

S.2.1 Fetal ultrasound

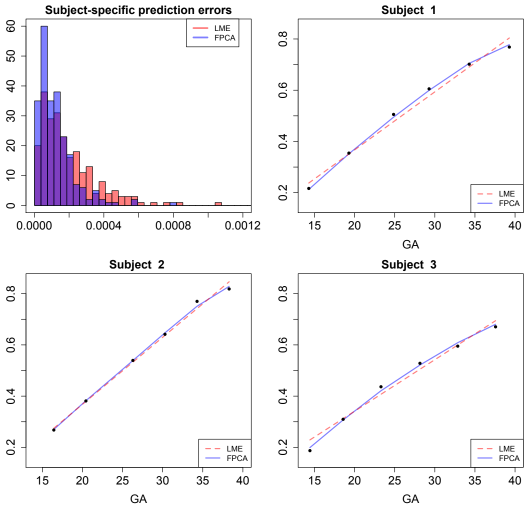

Figure S.2: Histograms of subject-specific square prediction errors and prediction of 3 subjects from the fPCA and LME models for the ultrasound measurements.

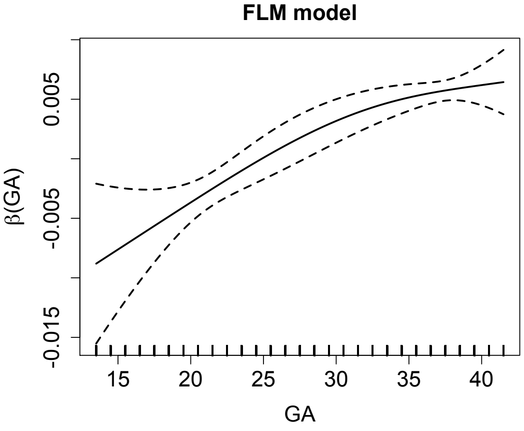

Figure S.3: Estimated coefficient function (solid line) for predicting birth weight. The dashed lines are point-wise 95% confidence bands.

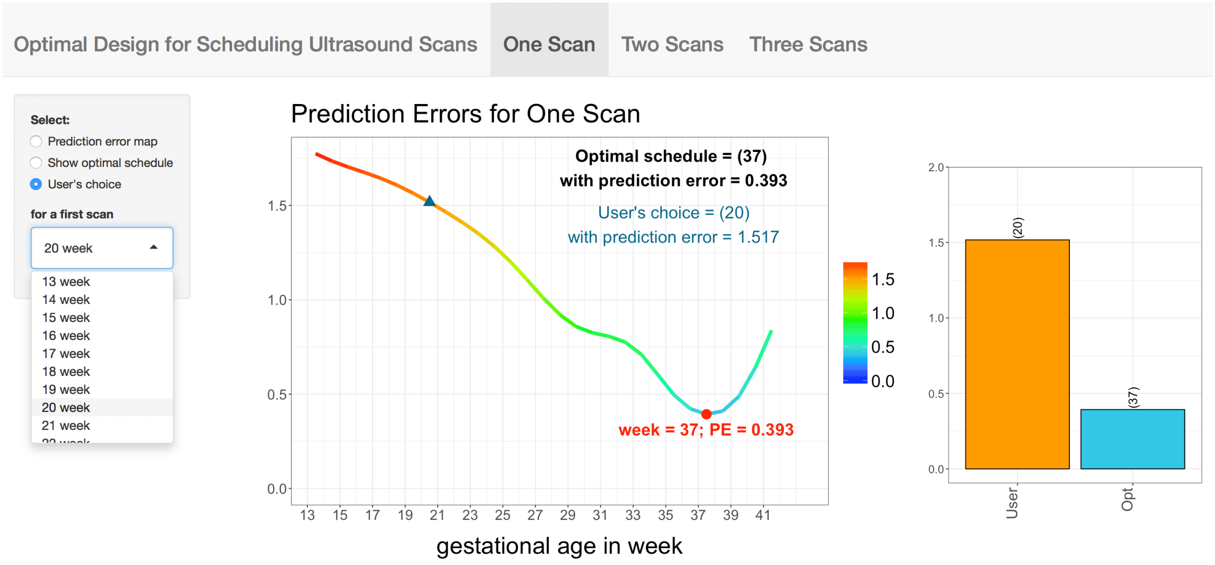

Figure S.4: Screenshot showing tab 1 of the interactive graphic with an objective function $\mathcal{M}$ evaluated at all possible candidate points.

S.2.2 Berkeley growth

We use the Berkeley growth data (Tuddenham and Snyder, 1954; Ramsay and Silverman, 2005) available in the R package *fda* (Ramsay et al., 2014) ctonains the heights of 39 boys and 54 girls at 31 different ages from age 1 to 18. We use the heights from age 1 to age 12 as the functional data and the heights at age 18 as the scalar response. We study the joint optimal design of recovering the height trajectory from age 1 to age 12 as well as predicting the height at age 18 for boys and girls, respectively. Figure S.5 shows the optimal design with 2 points: the most predictive 2 points for girls are age 3 and 11 while they are age 2 and 11 for boys.

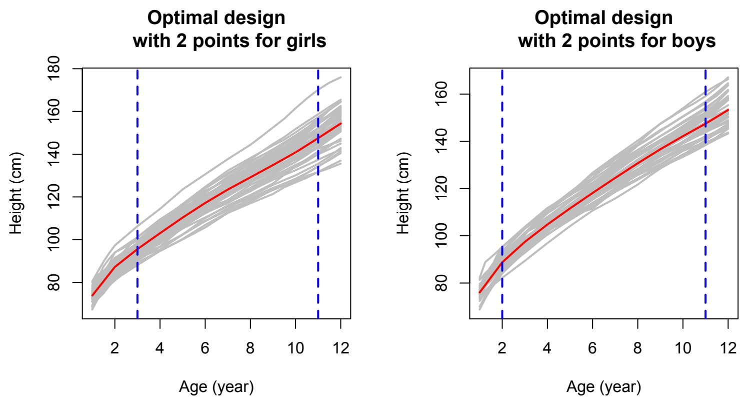

Figure S.5: Optimal design for the Berkeley growth data. The red curves are the estimated mean functions for boys and girls, respectively. The blue dashed lines are the selected optimal points.

S.3 Additional Results for the Simulation Study

S.3.1 Simulation Setting

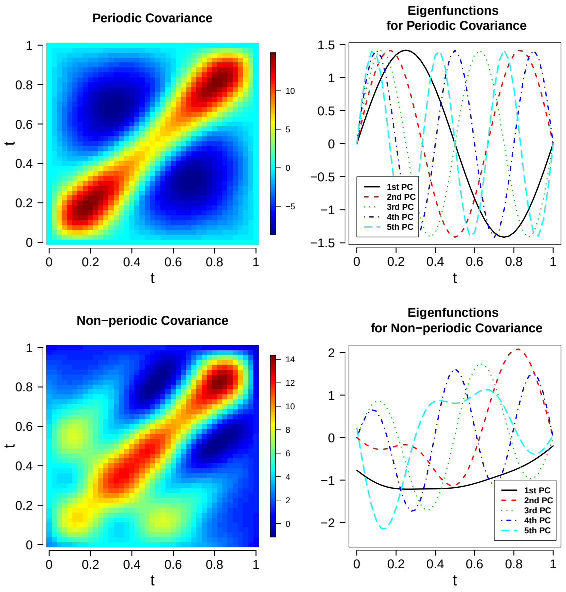

Figure S.6: Covariance functions (left panels) and corresponding eigenfunctions (right panels). The covariance is periodic if $r(s,t) = r(1-s,t)$.

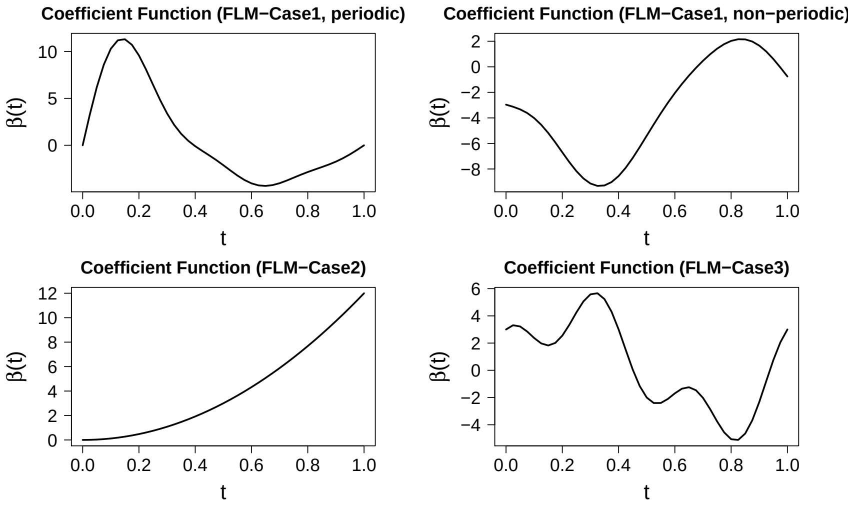

Figure S.7: Four choices of coefficient function $\beta(t)$ for the functional linear model (2) in Section 2.1.

S.3.2 Model Identifiability

The identifiability issue observed with covariance function of a periodic feature is illustrated in Figure S.8 and in Figure S.9.

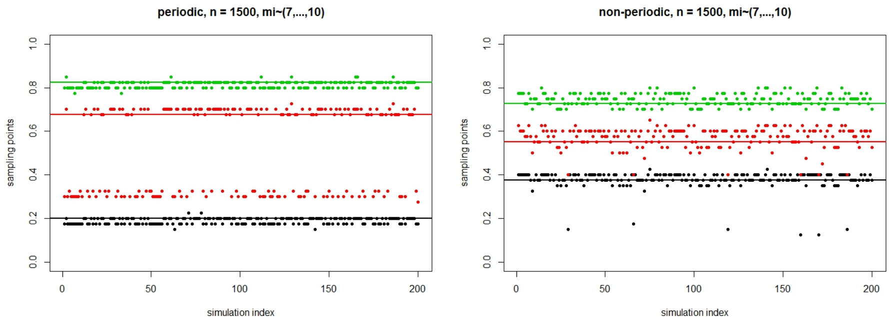

Figure S.8: Illustration of the identifiability issues; three horizontal lines indicate locations of three true optimal points $\mathbf{t}^*$ whereas solid dots indicate $N_{sim} = 200$ sets of three optimal points selected using the fPCA model with fixed $p = 3$.

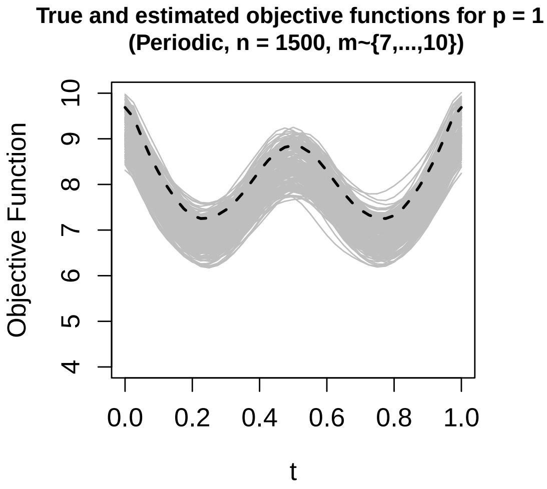

Figure S.9: Illustration of the identifiability issues; true objective function $\mathcal{M}(t)$ (dashed lines) and $N_{sim} = 200$ estimated objective functions $\widehat{\mathcal{M}}(t)$ (gray lines) for the periodic covariance.

S.3.3 Additional simulation results for the joint optimal design

Table S.1: Median of $\{\mathcal{M}\left(\widehat{\boldsymbol{t}}_{p,i_{sim}}\right) : i_{sim} = 1, \ldots, 200\}$ with the corresponding interquartile ranges (IQR) in parentheses for the case of periodic covariance.

	**Joint-Case1**		**Joint-Case2**		**Joint-Case3**	
periodic, $n = 400$, $m_i \sim \{3, 4, 5\}$						
	non-parametric	*parametric*	*non-parametric*	*parametric*	*non-parametric*	*parametric*
$p = 1$	1.21 (0.02)	13.54 (0.00)	1.21 (0.03)	2.00 (0.00)	1.33 (0.05)	2.00 (0.00)
$p = 2$	0.94 (0.03)	13.29 (0.44)	0.95 (0.05)	1.97 (0.09)	0.93 (0.02)	2.00 (0.04)
$p = 3$	0.77 (0.01)	13.10 (0.19)	0.78 (0.03)	1.91 (0.22)	0.79 (0.04)	1.95 (0.00)
$p = 4$	0.67 (0.02)	12.65 (0.86)	0.68 (0.02)	1.70 (0.19)	0.69 (0.03)	1.91 (0.06)
$p = 5$	0.59 (0.03)	11.76 (0.22)	0.60 (0.02)	1.67 (0.19)	0.60 (0.04)	1.79 (0.01)
periodic, $n = 400$, $m_i \sim \{7, \ldots, 10\}$						
	**Joint-Case1**		**Joint-Case2**		**Joint-Case3**	
	non-parametric	*parametric*	*non-parametric*	*parametric*	*non-parametric*	*parametric*
$p = 1$	1.20 (0.02)	2.00 (0.00)	1.20 (0.01)	2.00 (0.00)	1.30 (0.05)	2.00 (0.00)
$p = 2$	0.94 (0.01)	1.92 (0.08)	0.94 (0.01)	1.91 (0.09)	0.93 (0.01)	2.00 (0.00)
$p = 3$	0.77 (0.01)	1.92 (0.20)	0.77 (0.01)	1.91 (0.22)	0.77 (0.02)	1.95 (0.00)
$p = 4$	0.65 (0.02)	1.72 (0.00)	0.66 (0.02)	1.70 (0.00)	0.66 (0.02)	1.91 (0.00)
$p = 5$	0.58 (0.02)	1.51 (0.18)	0.58 (0.02)	1.47 (0.19)	0.58 (0.02)	1.79 (0.01)
periodic, $n = 800$, $m_i \sim \{3, 4, 5\}$						
	**Joint-Case1**		**Joint-Case2**		**Joint-Case3**	
	non-parametric	*parametric*	*non-parametric*	*parametric*	*non-parametric*	*parametric*
$p = 1$	1.20 (0.02)	2.00 (0.00)	1.20 (0.01)	2.00 (0.00)	1.30 (0.05)	2.00 (0.00)
$p = 2$	0.94 (0.01)	2.00 (0.08)	0.94 (0.01)	1.97 (0.09)	0.93 (0.01)	2.00 (0.00)
$p = 3$	0.77 (0.01)	1.92 (0.00)	0.77 (0.02)	1.91 (0.00)	0.77 (0.03)	1.95 (0.00)
$p = 4$	0.66 (0.03)	1.72 (0.17)	0.67 (0.03)	1.70 (0.19)	0.67 (0.03)	1.91 (0.02)
$p = 5$	0.58 (0.02)	1.69 (0.18)	0.59 (0.02)	1.67 (0.19)	0.59 (0.02)	1.79 (0.01)
periodic, $n = 800$, $m_i \sim \{7, \ldots, 10\}$						
	**Joint-Case1**		**Joint-Case2**		**Joint-Case3**	
	non-parametric	*parametric*	*non-parametric*	*parametric*	*non-parametric*	*parametric*
$p = 1$	1.20 (0.02)	2.00 (0.00)	1.20 (0.00)	2.00 (0.00)	1.30 (0.05)	2.00 (0.00)
$p = 2$	0.93 (0.01)	1.92 (0.06)	0.94 (0.01)	1.91 (0.09)	0.93 (0.00)	2.00 (0.00)
$p = 3$	0.76 (0.01)	1.92 (0.20)	0.77 (0.01)	1.91 (0.22)	0.76 (0.01)	1.95 (0.00)
$p = 4$	0.65 (0.01)	1.72 (0.00)	0.66 (0.01)	1.70 (0.00)	0.65 (0.01)	1.91 (0.00)
$p = 5$	0.57 (0.01)	1.51 (0.18)	0.58 (0.01)	1.47 (0.19)	0.57 (0.01)	1.79 (0.01)
periodic, $n = 1500$, $m_i \sim \{3, 4, 5\}$						
	**Joint-Case1**		**Joint-Case2**		**Joint-Case3**	
	non-parametric	*parametric*	*non-parametric*	*parametric*	*non-parametric*	*parametric*
$p = 1$	1.20 (0.02)	2.00 (0.00)	1.20 (0.01)	2.00 (0.00)	1.30 (0.05)	2.00 (0.00)
$p = 2$	0.93 (0.00)	1.92 (0.08)	0.94 (0.01)	1.91 (0.09)	0.93 (0.01)	2.00 (0.00)
$p = 3$	0.77 (0.01)	1.92 (0.00)	0.77 (0.01)	1.91 (0.00)	0.77 (0.02)	1.95 (0.00)
$p = 4$	0.65 (0.02)	1.72 (0.00)	0.66 (0.02)	1.70 (0.00)	0.66 (0.02)	1.91 (0.00)
$p = 5$	0.58 (0.02)	1.69 (0.18)	0.59 (0.02)	1.67 (0.19)	0.58 (0.02)	1.79 (0.01)
periodic, $n = 1500$, $m_i \sim \{7, \ldots, 10\}$						
	**Joint-Case1**		**Joint-Case2**		**Joint-Case3**	
	non-parametric	*parametric*	*non-parametric*	*parametric*	*non-parametric*	*parametric*
$p = 1$	1.20 (0.00)	2.00 (0.00)	1.20 (0.00)	2.00 (0.00)	1.30 (0.01)	2.00 (0.00)
$p = 2$	0.93 (0.00)	1.92 (0.00)	0.94 (0.01)	1.91 (0.00)	0.92 (0.00)	2.00 (0.00)
$p = 3$	0.76 (0.01)	1.92 (0.20)	0.77 (0.01)	1.91 (0.22)	0.76 (0.01)	1.95 (0.00)
$p = 4$	0.65 (0.01)	1.72 (0.00)	0.65 (0.01)	1.70 (0.00)	0.65 (0.01)	1.91 (0.00)
$p = 5$	0.57 (0.01)	1.51 (0.00)	0.57 (0.01)	1.47 (0.00)	0.57 (0.01)	1.79 (0.01)
11 equi-spaced	0.47		0.48		0.44	

Note: **Joint-Case1** indicates that the scalar responses are generated using $\beta(t)$ in FLM-Case1; similarly, **Joint-Case2** corresponds to FLM-Case2 and **Joint-Case3** to FLM-Case3. *non-parametric* and *parametric* refer to the covariance estimation using the fPCA and LME models, respectively.

Table S.2: Median of $\{\mathcal{M}\left(\widehat{\boldsymbol{t}}_{p,i_{sim}}\right) : i_{sim} = 1, \ldots, 200\}$ with the corresponding interquartile ranges (IQR) in parentheses for the case of non-periodic covariance.

	**Joint-Case1**		**Joint-Case2**		**Joint-Case3**	
	non-parametric	*parametric*	*non-parametric*	*parametric*	*non-parametric*	*parametric*
periodic, $n = 400$, $m_i \sim \{3, 4, 5\}$						
$p = 1$	1.36 (0.07)	1.69 (0.00)	1.46 (0.05)	1.71 (0.00)	1.57 (0.04)	1.84 (0.00)
$p = 2$	1.07 (0.08)	1.47 (0.00)	1.14 (0.10)	1.48 (0.22)	1.21 (0.08)	1.72 (0.00)
$p = 3$	0.92 (0.06)	1.32 (0.00)	0.95 (0.07)	1.47 (0.32)	1.04 (0.11)	1.64 (0.08)
$p = 4$	0.78 (0.07)	1.23 (0.00)	0.81 (0.06)	1.42 (0.33)	0.93 (0.11)	1.63 (0.05)
$p = 5$	0.68 (0.05)	1.17 (0.01)	0.72 (0.06)	1.34 (0.19)	0.83 (0.11)	1.58 (0.05)
periodic, $n = 400$, $m_i \sim \{7, \ldots, 10\}$						
	**Joint-Case1**		**Joint-Case2**		**Joint-Case3**	
	non-parametric	*parametric*	*non-parametric*	*parametric*	*non-parametric*	*parametric*
$p = 1$	1.33 (0.04)	1.69 (0.00)	1.46 (0.04)	1.71 (0.06)	1.56 (0.03)	1.84 (0.00)
$p = 2$	1.04 (0.02)	1.47 (0.00)	1.08 (0.04)	1.69 (0.00)	1.18 (0.02)	1.72 (0.00)
$p = 3$	0.89 (0.02)	1.32 (0.00)	0.92 (0.03)	1.64 (0.17)	1.00 (0.03)	1.72 (0.00)
$p = 4$	0.74 (0.02)	1.23 (0.00)	0.79 (0.02)	1.42 (0.12)	0.89 (0.03)	1.63 (0.00)
$p = 5$	0.65 (0.01)	1.17 (0.05)	0.69 (0.02)	1.34 (0.00)	0.78 (0.02)	1.59 (0.00)
periodic, $n = 800$, $m_i \sim \{3, 4, 5\}$						
	**Joint-Case1**		**Joint-Case2**		**Joint-Case3**	
	non-parametric	*parametric*	*non-parametric*	*parametric*	*non-parametric*	*parametric*
$p = 1$	1.33 (0.03)	1.69 (0.00)	1.45 (0.02)	1.71 (0.00)	1.56 (0.03)	1.84 (0.00)
$p = 2$	1.05 (0.03)	1.47 (0.00)	1.09 (0.06)	1.69 (0.22)	1.02 (0.05)	1.72 (0.00)
$p = 3$	0.90 (0.04)	1.32 (0.00)	0.93 (0.04)	1.64 (0.32)	1.01 (0.06)	1.64 (0.08)
$p = 4$	0.75 (0.03)	1.23 (0.00)	0.79 (0.03)	1.42 (0.31)	0.90 (0.06)	1.63 (0.05)
$p = 5$	0.66 (0.03)	1.17 (0.00)	0.70 (0.02)	1.34 (0.09)	0.79 (0.07)	1.59 (0.05)
periodic, $n = 800$, $m_i \sim \{7, \ldots, 10\}$						
	**Joint-Case1**		**Joint-Case2**		**Joint-Case3**	
	non-parametric	*parametric*	*non-parametric*	*parametric*	*non-parametric*	*parametric*
$p = 1$	1.32 (0.02)	1.69 (0.00)	1.45 (0.02)	1.71 (0.12)	1.55 (0.02)	1.84 (0.00)
$p = 2$	1.04 (0.02)	1.47 (0.00)	1.07 (0.01)	1.69 (0.00)	1.18 (0.01)	1.72 (0.00)
$p = 3$	0.88 (0.01)	1.32 (0.00)	0.90 (0.03)	1.64 (0.00)	0.99 (0.01)	1.72 (0.00)
$p = 4$	0.74 (0.02)	1.23 (0.00)	0.78 (0.01)	1.42 (0.00)	0.88 (0.02)	1.63 (0.00)
$p = 5$	0.64 (0.01)	1.17 (0.05)	0.68 (0.01)	1.34 (0.00)	0.78 (0.02)	1.59 (0.00)
periodic, $n = 1500$, $m_i \sim \{3, 4, 5\}$						
	**Joint-Case1**		**Joint-Case2**		**Joint-Case3**	
	non-parametric	*parametric*	*non-parametric*	*parametric*	*non-parametric*	*parametric*
$p = 1$	1.33 (0.02)	1.69 (0.00)	1.45 (0.02)	1.71 (0.00)	1.57 (0.04)	1.84 (0.00)
$p = 2$	1.04 (0.02)	1.47 (0.00)	1.08 (0.02)	1.69 (0.22)	1.23 (0.07)	1.72 (0.00)
$p = 3$	0.89 (0.02)	1.32 (0.00)	0.92 (0.03)	1.64 (0.32)	1.01 (0.03)	1.64 (0.08)
$p = 4$	0.74 (0.02)	1.23 (0.00)	0.79 (0.02)	1.42 (0.26)	0.90 (0.02)	1.63 (0.05)
$p = 5$	0.65 (0.02)	1.17 (0.00)	0.69 (0.02)	1.34 (0.09)	0.80 (0.03)	1.58 (0.05)
periodic, $n = 1500$, $m_i \sim \{7, \ldots, 10\}$						
	**Joint-Case1**		**Joint-Case2**		**Joint-Case3**	
	non-parametric	*parametric*	*non-parametric*	*parametric*	*non-parametric*	*parametric*
$p = 1$	1.32 (0.01)	1.69 (0.00)	1.45 (0.03)	1.67 (0.15)	1.55 (0.01)	1.84 (0.00)
$p = 2$	1.04 (0.01)	1.47 (0.00)	1.07 (0.01)	1.69 (0.00)	1.17 (0.01)	1.72 (0.00)
$p = 3$	0.88 (0.01)	1.32 (0.00)	0.89 (0.01)	1.64 (0.00)	0.99 (0.02)	1.72 (0.00)
$p = 4$	0.73 (0.01)	1.23 (0.00)	0.78 (0.01)	1.42 (0.00)	0.88 (0.02)	1.63 (0.00)
$p = 5$	0.64 (0.02)	1.17 (0.05)	0.68 (0.01)	1.34 (0.00)	0.77 (0.02)	1.59 (0.00)
11 equi-spaced	0.46		0.48		0.59	

Note: **Joint-Case1** indicates that the scalar responses are generated using $\beta(t)$ in FLM-Case1; similarly, **Joint-Case2** corresponds to FLM-Case2 and **Joint-Case3** to FLM-Case3. *non-parametric* and *parametric* refer to the covariance estimation using the fPCA and LME models, respectively.

Table S.3: Median of absolute relative errors, $\{\text{ARE}_{p,i_{sim}} : i_{sim} = 1, \ldots, 200\}$ and the corresponding interquartile ranges (IQR) in parentheses for the case of the non-periodic covariance case.

		Joint-Case1		Joint-Case2		Joint-Case3	
$p = 3$							
		non-parametric	*parametric*	*non-parametric*	*parametric*	*non-parametric*	*parametric*
$n = 400$	$m_i \sim \{3, 4, 5\}$	0.062 (0.072)	0.519 (0.000)	0.070 (0.083)	0.646 (0.363)	0.061 (0.110)	0.672 (0.082)
	$m_i \sim \{7, \ldots, 10\}$	0.020 (0.022)	0.519 (0.000)	0.032 (0.033)	0.835 (0.188)	0.019 (0.029)	0.754 (0.000)
$n = 800$	$m_i \sim \{3, 4, 5\}$	0.034 (0.044)	0.519 (0.000)	0.040 (0.050)	0.835 (0.363)	0.030 (0.066)	0.672 (0.082)
	$m_i \sim \{7, \ldots, 10\}$	0.014 (0.017)	0.519 (0.000)	0.010 (0.030)	0.835 (0.000)	0.012 (0.014)	0.754 (0.000)
$n = 1500$	$m_i \sim \{3, 4, 5\}$	0.021 (0.023)	0.519 (0.000)	0.026 (0.038)	0.835 (0.363)	0.033 (0.035)	0.672 (0.082)
	$m_i \sim \{7, \ldots, 10\}$	0.009 (0.014)	0.519 (0.000)	0.003 (0.010)	0.835 (0.000)	0.009 (0.016)	0.754 (0.000)
$p = 4$							
		**Joint-Case1**		**Joint-Case2**		**Joint-Case3**	
		non-parametric	*parametric*	*non-parametric*	*parametric*	*non-parametric*	*parametric*
$n = 400$	$m_i \sim \{3, 4, 5\}$	0.073 (0.095)	0.696 (0.000)	0.053 (0.072)	0.846 (0.431)	0.082 (0.125)	0.892 (0.061)
	$m_i \sim \{7, \ldots, 10\}$	0.020 (0.025)	0.696 (0.000)	0.020 (0.023)	0.846 (0.153)	0.030 (0.034)	0.892 (0.000)
$n = 800$	$m_i \sim \{3, 4, 5\}$	0.037 (0.036)	0.696 (0.000)	0.031 (0.033)	0.846 (0.396)	0.044 (0.064)	0.892 (0.061)
	$m_i \sim \{7, \ldots, 10\}$	0.015 (0.021)	0.696 (0.000)	0.015 (0.016)	0.846 (0.000)	0.024 (0.021)	0.892 (0.000)
$n = 1500$	$m_i \sim \{3, 4, 5\}$	0.022 (0.029)	0.696 (0.000)	0.021 (0.026)	0.846 (0.336)	0.041 (0.027)	0.892 (0.061)
	$m_i \sim \{7, \ldots, 10\}$	0.013 (0.018)	0.696 (0.000)	0.006 (0.013)	0.846 (0.000)	0.021 (0.025)	0.892 (0.000)
$p = 5$							
		**Joint-Case1**		**Joint-Case2**		**Joint-Case3**	
		non-parametric	*parametric*	*non-parametric*	*parametric*	*non-parametric*	*parametric*
$n = 400$	$m_i \sim \{3, 4, 5\}$	0.071 (0.084)	0.857 (0.020)	0.063 (0.082)	0.991 (0.286)	0.097 (0.145)	1.075 (0.064)
	$m_i \sim \{7, \ldots, 10\}$	0.025 (0.022)	0.857 (0.081)	0.019 (0.029)	0.991 (0.000)	0.025 (0.028)	1.092 (0.000)
$n = 800$	$m_i \sim \{3, 4, 5\}$	0.039 (0.043)	0.857 (0.000)	0.037 (0.036)	0.991 (0.132)	0.045 (0.088)	1.092 (0.064)
	$m_i \sim \{7, \ldots, 10\}$	0.020 (0.024)	0.857 (0.081)	0.011 (0.014)	0.991 (0.000)	0.022 (0.025)	1.092 (0.000)
$n = 1500$	$m_i \sim \{3, 4, 5\}$	0.025 (0.026)	0.857 (0.000)	0.020 (0.028)	0.991 (0.132)	0.058 (0.040)	1.075 (0.064)
	$m_i \sim \{7, \ldots, 10\}$	0.020 (0.024)	0.857 (0.081)	0.008 (0.009)	0.991 (0.000)	0.019 (0.028)	1.092 (0.000)

Note: **Joint-Case1** indicates that the scalar responses are generated using $\beta(t)$ in FLM-Case1; similarly, **Joint-Case2** corresponds to FLM-Case2 and **Joint-Case3** to FLM-Case3. *non-parametric* and *parametric* refer to the covariance estimation using the fPCA and LME models, respectively.

Table S.4: Proportion of selected number of points being equal to 3 for the case of the non-periodic covariance

		**Joint-Case1**	**Joint-Case2**	**Joint-Case3**
$n = 400$	$m_i \sim \{3, 4, 5\}$	0.75	0.90	0.98
	$m_i \sim \{7, \ldots, 10\}$	0.78	0.89	0.98
$n = 800$	$m_i \sim \{3, 4, 5\}$	0.81	0.88	0.99
	$m_i \sim \{7, \ldots, 10\}$	0.82	0.87	1.00
$n = 1500$	$m_i \sim \{3, 4, 5\}$	0.82	0.84	0.99
	$m_i \sim \{7, \ldots, 10\}$	0.90	0.92	0.99

Note: **Joint-Case1** indicates that the scalar responses are generated using $\beta(t)$ in FLM-Case1; similarly, **Joint-Case2** corresponds to FLM-Case2 and **Joint-Case3** to FLM-Case3.

S.3.4 Simulation results with data generated from LME models

We conduct an additional simulation study to investigate the effect of parametric and nonparametric modeling of the covariance function $r(s,t)$ on estimation of optimal sampling points when data are generated from a linear mixed effects model.

Specifically we generate each of $N_{sim} = 200$ MC samples, $\{(W_{ij}, t_{ij}) : i = 1, \ldots, n \text{ and } j = 1, \ldots, m_i\}$, from the LME model, $W_{ij} = \mu(t_{ij}) + b_{i0} + b_{i1}t_{ij} + \epsilon_{ij}$ with $\mu(t) = 0, \forall t$. Pairs of subject-specific random intercept and slope, (b_{i0}, b_{i1}), are independently drawn from multivariate normal distribution with mean zeros, variances (τ_0^2, τ_1^2), and correlation ρ ; we set $\tau_0^2 = 0.25^2$ and $\tau_1^2 = 1$, and we consider two correlation values, $\rho = -0,2$ and $\rho = -0,9$. White noises ϵ_{ij} are drawn independently from a normal distribution with mean zero and variance $\sigma_\epsilon^2 = 1$. The number of repeated measures per subject, m_i, are independently drawn from Uniform$\{3, 4, 5\}$. For subject i, m_i number of sampling points t_{ij} are drawn independently from the uniform distribution in the unit interval.

For the FLM framework, in addition to W_{ij}'s and t_{ij}'s, we generate a scalar response Y_i from the following model, $Y_i = \int(b_{i0} + b_{i1}t)\beta(t)dt + e_i$ with the same random intercept b_{i0} and slope b_{i1} used to generate W_{ij}'s. We use two different coefficient functions $\beta(t)$:

FLM-Case-S1: $\beta(t) = 1$.

FLM-Case-S2: $\beta(t) = 1 + 2t$.

Random errors e_i's are independently drawn from a normal distribution with mean zero and variance $\sigma_e^2 = 1$. Lastly we consider sample sizes $n = 800$ and $n = 1500$.

From Table S.5 we observe that five selected optimal points achieve the same level of prediction accuracy as 11 equi-spaced points when correlation $\rho = -0.2$; whereas four selected optimal points achieve the same error level as 11 equi-spaced points with strong correlation $\rho = -0.9$. Based on the results shown in Tables S.5 and S.6, when the underlying covariance of the data is parametric the proposed method of estimating optimal sampling points performs equally well across all the settings, regardless of using either *parametric* or *non-parametric* modeling of the covariance function, respectively. In summary the proposed method with *non-parametric* modeling using fPCA performs well on data with both simple and complex covariance.

Table S.5: Median of $\{\mathcal{M}(\widehat{\boldsymbol{t}}_{p,i_{sim}}) : i_{sim} = 1, \dots, 200\}$ with the corresponding interquartile ranges (IQR) in parentheses obtained using MC samples generated from LME.

$\rho = -0.2$, $n = 800$, $m_i \sim \{3,4,5\}$						
	**LME**		**FLM-Case-S1**		**FLM-Case-S2**	
	non-parametric	*parametric*	*non-parametric*	*parametric*	*non-parametric*	*parametric*
$p = 1$	0.19 (0.00)	0.19 (0.00)	0.14 (0.00)	0.14 (0.00)	0.73 (0.00)	0.73 (0.00)
$p = 2$	0.14 (0.00)	0.14 (0.00)	0.10 (0.00)	0.10 (0.00)	0.51 (0.00)	0.51 (0.00)
$p = 3$	0.11 (0.00)	0.11 (0.00)	0.08 (0.00)	0.08 (0.00)	0.40 (0.00)	0.40 (0.00)
$p = 4$	0.09 (0.00)	0.09 (0.00)	0.07 (0.00)	0.07 (0.00)	0.33 (0.00)	0.33 (0.00)
$p = 5$	0.08 (0.00)	0.08 (0.00)	0.06 (0.00)	0.06 (0.00)	0.29 (0.00)	0.29 (0.00)
11 equi-spaced	0.08		0.06		0.29	
$\rho = -0.2$, $n = 1500$, $m_i \sim \{3,4,5\}$						
	**LME**		**FLM-Case-S1**		**FLM-Case-S2**	
	non-parametric	*parametric*	*non-parametric*	*parametric*	*non-parametric*	*parametric*
$p = 1$	0.19 (0.00)	0.19 (0.00)	0.14 (0.00)	0.14 (0.00)	0.73 (0.00)	0.73 (0.00)
$p = 2$	0.14 (0.00)	0.14 (0.00)	0.10 (0.00)	0.10 (0.00)	0.51 (0.00)	0.51 (0.00)
$p = 3$	0.11 (0.00)	0.11 (0.00)	0.08 (0.00)	0.08 (0.00)	0.40 (0.00)	0.40 (0.00)
$p = 4$	0.09 (0.00)	0.09 (0.00)	0.07 (0.00)	0.07 (0.00)	0.33 (0.00)	0.33 (0.00)
$p = 5$	0.08 (0.00)	0.08 (0.00)	0.06 (0.00)	0.06 (0.00)	0.29 (0.00)	0.29 (0.00)
11 equi-spaced	0.08		0.06		0.29	
$\rho = -0.9$, $n = 800$, $m_i \sim \{3,4,5\}$						
	**LME**		**FLM-Case-S1**		**FLM-Case-S2**	
	non-parametric	*parametric*	*non-parametric*	*parametric*	*non-parametric*	*parametric*
$p = 1$	0.11 (0.00)	0.11 (0.00)	0.06 (0.00)	0.06 (0.00)	0.36 (0.00)	0.36 (0.00)
$p = 2$	0.08 (0.00)	0.08 (0.00)	0.04 (0.00)	0.04 (0.00)	0.27 (0.00)	0.27 (0.00)
$p = 3$	0.07 (0.00)	0.07 (0.00)	0.04 (0.00)	0.04 (0.00)	0.22 (0.00)	0.22 (0.00)
$p = 4$	0.06 (0.00)	0.06 (0.00)	0.03 (0.00)	0.03 (0.00)	0.19 (0.00)	0.19 (0.00)
$p = 5$	0.05 (0.00)	0.05 (0.00)	0.03 (0.00)	0.03 (0.00)	0.16 (0.00)	0.16 (0.00)
11 equi-spaced	0.06		0.03		0.20	
$\rho = -0.9$, $n = 1500$, $m_i \sim \{3,4,5\}$						
	**LME**		**FLM-Case-S1**		**FLM-Case-S2**	
	non-parametric	*parametric*	*non-parametric*	*parametric*	*non-parametric*	*parametric*
$p = 1$	0.11 (0.00)	0.11 (0.00)	0.06 (0.00)	0.06 (0.00)	0.36 (0.00)	0.36 (0.00)
$p = 2$	0.08 (0.00)	0.08 (0.00)	0.04 (0.00)	0.04 (0.00)	0.27 (0.00)	0.27 (0.00)
$p = 3$	0.07 (0.00)	0.07 (0.00)	0.04 (0.00)	0.04 (0.00)	0.22 (0.00)	0.22 (0.00)
$p = 4$	0.06 (0.00)	0.06 (0.00)	0.03 (0.00)	0.03 (0.00)	0.19 (0.00)	0.19 (0.00)
$p = 5$	0.05 (0.00)	0.05 (0.00)	0.03 (0.00)	0.03 (0.00)	0.16 (0.00)	0.16 (0.00)
11 equi-spaced	0.06		0.03		0.20	

Note: *non-parametric* and *parametric* in the table refer to covariance estimation using the fPCA and LME models, respectively.

Table S.6: Median of absolute relative errors, $\{\text{ARE}_{p,i_{sim}} : i_{sim} = 1, \ldots, 200\}$ and the corresponding interquartile ranges (IQR) in parentheses obtained using MC samples generated from LME.

$p = 3$			LME		FLM-Case-S1		FLM-Case-S2	
			non-parametric	*parametric*	*non-parametric*	*parametric*	*non-parametric*	*parametric*
$\rho = -0.2$	$n = 800$	$m_i \sim \{3, 4, 5\}$	0.00 (0.00)	0.00 (0.00)	0.00 (0.00)	0.00 (0.00)	0.00 (0.00)	0.00 (0.00)
$\rho = -0.2$	$n = 1500$	$m_i \sim \{3, 4, 5\}$	0.00 (0.00)	0.00 (0.00)	0.00 (0.00)	0.00 (0.00)	0.00 (0.00)	0.00 (0.00)
$\rho = -0.9$	$n = 800$	$m_i \sim \{3, 4, 5\}$	0.00 (0.00)	0.00 (0.00)	0.00 (0.00)	0.00 (0.00)	0.00 (0.00)	0.00 (0.00)
$\rho = -0.9$	$n = 1500$	$m_i \sim \{3, 4, 5\}$	0.00 (0.00)	0.00 (0.00)	0.00 (0.00)	0.00 (0.00)	0.00 (0.00)	0.00 (0.00)

$p = 4$			LME		FLM-Case-S1		FLM-Case-S2	
			non-parametric	*parametric*	*non-parametric*	*parametric*	*non-parametric*	*parametric*
$\rho = -0.2$	$n = 800$	$m_i \sim \{3, 4, 5\}$	0.00 (0.00)	0.00 (0.00)	0.00 (0.02)	0.00 (0.00)	0.00 (0.00)	0.00 (0.00)
$\rho = -0.2$	$n = 1500$	$m_i \sim \{3, 4, 5\}$	0.00 (0.00)	0.00 (0.00)	0.00 (0.02)	0.00 (0.00)	0.00 (0.00)	0.00 (0.00)
$\rho = -0.9$	$n = 800$	$m_i \sim \{3, 4, 5\}$	0.00 (0.00)	0.00 (0.00)	0.00 (0.03)	0.00 (0.00)	0.00 (0.00)	0.00 (0.00)
$\rho = -0.9$	$n = 1500$	$m_i \sim \{3, 4, 5\}$	0.00 (0.00)	0.00 (0.00)	0.00 (0.05)	0.00 (0.00)	0.00 (0.00)	0.00 (0.00)

$p = 5$			LME		FLM-Case-S1		FLM-Case-S2	
			non-parametric	*parametric*	*non-parametric*	*parametric*	*non-parametric*	*parametric*
$\rho = -0.2$	$n = 800$	$m_i \sim \{3, 4, 5\}$	0.00 (0.02)	0.00 (0.00)	0.00 (0.07)	0.00 (0.00)	0.00 (0.01)	0.00 (0.00)
$\rho = -0.2$	$n = 1500$	$m_i \sim \{3, 4, 5\}$	0.00 (0.00)	0.00 (0.00)	0.00 (0.05)	0.00 (0.00)	0.00 (0.01)	0.00 (0.00)
$\rho = -0.9$	$n = 800$	$m_i \sim \{3, 4, 5\}$	0.00 (0.00)	0.00 (0.00)	0.00 (0.12)	0.00 (0.00)	0.00 (0.00)	0.00 (0.00)
$\rho = -0.9$	$n = 1500$	$m_i \sim \{3, 4, 5\}$	0.00 (0.00)	0.00 (0.00)	0.00 (0.08)	0.00 (0.00)	0.00 (0.00)	0.00 (0.00)

Note: *non-parametric* and *parametric* in the table refer to covariance estimation using the fPCA and LME models, respectively.

S.3.5 Simulation results on uncertainty of selected optimal sampling points

Table S.7: Median of third quartile of bootstrap absolute relative errors $\{ARE^{(b)}_{p,i_{\text{sim}}} : b = 1, \ldots, 100\}$ for $i_{\text{sim}} = 1, \ldots, 200$

		$p = 3$	$p = 4$	$p = 5$
n = 400	$m_i \sim \{3, 4, 5\}$	0.101	0.135	0.151
	$m_i \sim \{7, \ldots, 10\}$	0.058	0.102	0.108
n = 800	$m_i \sim \{3, 4, 5\}$	0.069	0.106	0.117
	$m_i \sim \{7, \ldots, 10\}$	0.042	0.080	0.086
n = 1500	$m_i \sim \{3, 4, 5\}$	0.044	0.087	0.091
	$m_i \sim \{7, \ldots, 10\}$	0.027	0.061	0.073

Table S.8: Median of 90% percentile of bootstrap absolute relative errors $\{ARE^{(b)}_{p,i_{\text{sim}}} : b = 1, \ldots, 100\}$ for $i_{\text{sim}} = 1, \ldots, 200$

		$p = 3$	$p = 4$	$p = 5$
n = 400	$m_i \sim \{3, 4, 5\}$	0.158	0.185	0.199
	$m_i \sim \{7, \ldots, 10\}$	0.102	0.130	0.142
n = 800	$m_i \sim \{3, 4, 5\}$	0.107	0.135	0.150
	$m_i \sim \{7, \ldots, 10\}$	0.068	0.110	0.115
n = 1500	$m_i \sim \{3, 4, 5\}$	0.073	0.112	0.122
	$m_i \sim \{7, \ldots, 10\}$	0.049	0.093	0.096

References

Ramsay, J. and B. Silverman (2005). *Functional data analysis.* New York: Springer.

Ramsay, J., H. Wickham, S. Graves, and G. Hooker (2014). *fda: Functional Data Analysis.*

Tuddenham, R. and M. Snyder (1954). Physical growth of california boys and girls from birth to age 18. *University of California Publications in Child Development 1*, 183 – 364.
